# In-depth genome characterization of a Brazilian common bean core collection using DArTseq high-density SNP genotyping

**DOI:** 10.1186/s12864-017-3805-4

**Published:** 2017-05-30

**Authors:** Paula A. M. R. Valdisser, Wendell J. Pereira, Jâneo E. Almeida Filho, Bárbara S. F. Müller, Gesimária R. C. Coelho, Ivandilson P. P. de Menezes, João P. G. Vianna, Maria I. Zucchi, Anna C. Lanna, Alexandre S. G. Coelho, Jaison P. de Oliveira, Alessandra da Cunha Moraes, Claudio Brondani, Rosana P. Vianello

**Affiliations:** 1 Embrapa Arroz e Feijão (CNPAF), Santo Antônio de Goiás, Goiânia, GO Brazil; 2 Programa de Pós-Graduação em Biologia Molecular, Universidade de Brasília (UnB), Brasília, DF Brazil; 3 Universidade Estadual do Norte Fluminense Darcy Ribeiro (UENF), Campos dos Goytacazes, Rio de Janeiro, RJ Brazil; 4 Laboratório de Genética e Biologia Molecular, Departamento de Biologia, Instituto Federal Goiano (IF Goiano), Urutaí, GO Brazil; 5 Programa de Pós-Graduação em Genética e Biologia Molecular, Universidade Estadual de Campinas (UNICAMP), Campinas, SP Brazil; 6 Escola de Agronomia, Universidade Federal de Goiás (UFG), Goiânia, GO Brazil

**Keywords:** *Phaseolus vulgaris* L, Diversity arrays technology, Diversity analysis, Linkage disequilibrium, Loci under selection

## Abstract

**Background:**

Common bean is a legume of social and nutritional importance as a food crop, cultivated worldwide especially in developing countries, accounting for an important source of income for small farmers. The availability of the complete sequences of the two common bean genomes has dramatically accelerated and has enabled new experimental strategies to be applied for genetic research. DArTseq has been widely used as a method of SNP genotyping allowing comprehensive genome coverage with genetic applications in common bean breeding programs.

**Results:**

Using this technology, 6286 SNPs (1 SNP/86.5 Kbp) were genotyped in genic (43.3%) and non-genic regions (56.7%). Genetic subdivision associated to the common bean gene pools (K = 2) and related to grain types (K = 3 and K = 5) were reported. A total of 83% and 91% of all SNPs were polymorphic within the Andean and Mesoamerican gene pools, respectively, and 26% were able to differentiate the gene pools. Genetic diversity analysis revealed an average *H*_*E*_ of 0.442 for the whole collection, 0.102 for Andean and 0.168 for Mesoamerican gene pools (*F*_*ST*_ = 0.747 between gene pools), 0.440 for the group of cultivars and lines, and 0.448 for the group of landrace accessions (*F*_*ST*_ = 0.002 between cultivar/line and landrace groups). The SNP effects were predicted with predominance of impact on non-coding regions (77.8%). SNPs under selection were identified within gene pools comparing landrace and cultivar/line germplasm groups (Andean: 18; Mesoamerican: 69) and between the gene pools (59 SNPs), predominantly on chromosomes 1 and 9. The LD extension estimate corrected for population structure and relatedness (r^2^_SV_) was ~ 88 kbp, while for the Andean gene pool was ~ 395 kbp, and for the Mesoamerican was ~ 130 kbp.

**Conclusions:**

For common bean, DArTseq provides an efficient and cost-effective strategy of generating SNPs for large-scale genome-wide studies. The DArTseq resulted in an operational panel of 560 polymorphic SNPs in linkage equilibrium, providing high genome coverage. This SNP set could be used in genotyping platforms with many applications, such as population genetics, phylogeny relation between common bean varieties and support to molecular breeding approaches.

**Electronic supplementary material:**

The online version of this article (doi:10.1186/s12864-017-3805-4) contains supplementary material, which is available to authorized users.

## Background

It is estimated that approximately 150 plant species are grown directly for human consumption or animal feed worldwide, and 30 of them contribute to 95% of the calories and protein in the human diet [1]. Legumes, along with grasses, are the main source of human food [2]. Among legumes with edible dry seeds (pulses), over 80 species are widely cultivated, including the common bean, *Phaseolus vulgaris* L. [3]. Common bean is a very important crop for food security and sustainable agriculture. This species is considered the most important grain legume available for human consumption [4], being cultivated in 126 countries with an annual planted area estimated to be 30.6 million hectares [5] and representing 37% of all legumes consumed in the world. To ensure the preservation of the extensive genetic diversity of common bean, national and international gene banks were created. The International Common Bean Gene Bank at CIAT (International Center for Tropical Agriculture, Colombia) has more than 37,000 accessions, of which approximately 90% are cultivated *Phaseolus vulgaris* varieties [6]. In Brazil, the gene bank at Embrapa Rice and Beans has ~17,345 accessions, of which approximately 3.5% (~600 accessions) were selected to compose the core collection (acronym CONFE), which is made up of three strata: a) landraces from Brazil; b) cultivars/lines improved in Brazil; and c) introduced cultivars/lines, all of Andean and Mesoamerican origin. The seed samples are publicly available for research institutions in Brazil and abroad and are stored at Global Seeds Banking of Svalbard, located in Longyearbyen, Norway.

There is consensus regarding the predominant genetic structure of common bean in the Andean and Mesoamerican gene pools [7, 8], due to a divergence estimated to have occurred since 165,000 years ago [9]. Genes related to agronomic traits of great interest to current breeding programs, such as flowering, plant height, and nitrogen metabolism, were identified as being under selection during the domestication process [9]. The common bean landraces from Brazil, a secondary center of domestication, are adapted to diverse soil and climate conditions and present broad genetic diversity [10]. It is expected that several adaptive mechanisms selected over generations of domestication remain unknown [11] and can be used as an important source of useful genes for breeding programs [12]. A large proportion of plant genetic resources remains unexplored [13]. This situation is changing due to efforts in breeding programs to increase the available genetic diversity among the set of genitors used in crosses [14–16]. Through pre-breeding programs, work to identify favorable alleles of genes related to important agronomic traits in wild germplasm and landraces, with subsequent incorporation into improved crops, has been reviewed [17]. The availability of common bean reference genomes [9, 18], in addition to predicted functions for thousands of genes, extends the possibilities for marker-assisted selection, and to increase the efficiency of genetic breeding programs [19].

Molecular markers have been very helpful in efforts to detect gaps and redundancies in germplasm collections [20], to elucidate the genetic diversity in both wild germplasm [21] and in landraces and cultivars/lines [10, 22], to explore the effects of selection in the domestication process and to evaluate the dynamics of gene flow and genetic structure due to geographic distribution [23, 24]. Many of these studies were conducted using SSR markers [25–29]. In recent years, SNP markers have been increasingly developed and applied in common bean genetic analysis [29–31]. Based on 131 SNPs, Rodriguez et al. [16] analyzed a set of 577 wild and domesticated common bean accessions, drew conclusions about the genetic structure along the domestication sites and identified geographic regions that were hotspots of genetic diversity. More recently, a 6000 SNP chip was developed (BARCBean6K_3) and successfully used in linkage and genome-wide association mapping studies [32, 33].

High-density genotyping, combining genome complexity reduction with next-generation sequencing (NGS), allows the identification of an almost unlimited number of SNPs for any species at low cost. The strategies of restriction site-associated DNA sequencing (RADseq) [34] and genotyping by sequencing (GBS) [35] allow researchers to identify and genotype thousands of SNPs in several plant species, including common bean [31, 36]. The Diversity Arrays Technology methodology (DArT), also based on genome complexity reduction and SNP detection through hybridization of PCR fragments [37], has been used in the construction of dense linkage maps, mapping quantitative trait loci (QTL), genome-wide association studies (GWAS), and studies of genetic diversity and population structure [38–40]. In legumes, DArT markers were used to detect QTLs associated with resistance to angular leaf spot and genetic diversity studies [41, 42]. More recently, the application of DArT technology was modified to incorporate the advantages of the genotyping by sequencing approach (DArTseq™) [20, 43, 44].

In this study, DArTseq derived SNPs were used for the genetic analysis of a common bean germplasm collection of Andean and Mesoamerican origin, being each origin further stratified into cultivar/line and landrace groups. This study also made advances in the detection and characterization of genomic regions with signals of selection imposed by the domestication and breeding of common bean in Brazil. In addition, a set of SNPs with high discriminatory value between gene pools, as well as between groups (landraces and cultivars/lines) within gene pools, was proposed for routine use for the characterization of gene bank accessions and in breeding programs.

## Methods

### Plant material

A total of 188 common bean accessions, including 91 landraces and 97 Brazilian and international cultivars/lines belonging to the Andean and Mesoamerican gene pools, were used (Additional file 1). The accessions were planted in a greenhouse and multiplied via selfing in order to ensure homogeneity for genetic analysis. DNA from individual plants was extracted using the Invisorb Spin Plant Mini Kit (Stratec Molecular, Berlin, Germany), followed by shipment to a DArTseq analysis facility (DArT Pty Ltd., Bruce, Australia).

### Genotyping using DArTseq

DArTseq™ represents a combination of DArT complexity reduction methods, based on methyl filtration, and next-generation sequencing platforms [45]. The technology was optimized for common bean considering both the size of the representation and the fraction of the genome selected for analysis. The complexity reduction method was based on *PstI-MseI*. DNA samples were processed before and after sequencing as described by Sánchez-Sevilla et al. [44]. The amplification products were sequenced on the Illumina HiSeq2000 platform. Approximately 2,000,000 sequences per barcode/sample were identified and used in marker calling. Identical sequences were collapsed into *fastqcall* files. These files were used in the secondary pipeline for DArT PL’s proprietary SNP-calling algorithms (DArTsoft-seq). The DArTseq quality markers were determined by the parameters “reproducibility” (percentage of technical replicate pairs scoring identically for a given marker) and “call-rate” (percentage of samples for which a given marker was scored”) [46].

### Structural and functional characterization of SNPs

Genomic regions flanking SNPs were aligned against the reference genome of *P. vulgaris* v 1.0 [9] using BLASTN with an e-value ≤ 1.0e-25 [47]. Annotation and prediction of effect were performed using the SnpEff v 4.2 [48] based on the Phytozome database [49]. The SNP predicted effects were categorized by impact, as high (disruptive impact on the protein); moderate (non-synonymous substitution); low (synonymous substitution); modifier (with impact on non-coding regions).

SNPs with putative effects predicted to be moderate or high were functionally annotated using the Blast2GO tool v 3.2 [50] and characterized using Gene Ontology terms (Consortium 2015) [51]. KEGG (available in Blast2GO v 3.2) provided the Enzyme Code (EC) for metabolic pathways. The Integrative Genomics Viewer (IGV) [52] was used for visual inspection and gene models construction.

### Analysis of population genetics structure

The genetic structure, based on the Bayesian clustering approach, was implemented by Structure v 2.3.4 [53]. This analysis was conducted using 580 SNPs in linkage equilibrium (LE; r^2^ < 0.5) identified using Golden Helix SNP & Variation Suite v 8 (Golden Helix Inc., Bozeman, MT, USA) through the LD Prunning command. A population number (K) ranging from 1 to 20, with 20 interactions each, was assumed. The admixture model was applied using a 500,000 burn-in periods followed by 1,000,000 Markov Chain Monte Carlo (MCMC) replications. The most likely K was determined, as proposed by [54] using Structure Harvester v 0.6.93 [55], followed by analysis with CLUMPP v 1.1.2 [56]. The organization chart was generated in R v 3.1.3 [57]. Discriminant Analysis of Principal Components (DAPC) [58] was performed using the Adegenet package for R [57, 59] to provide further support for the identified population groups. The dendrogram was constructed using the neighbor joining (NJ) method implemented by Mega v 5 [60], based on a matrix calculated by Simple Matching Dissimilarity with 1,000 bootstrap interactions (Darwin 6.0.10) [61]. The Analysis of genetic diversity was performed in GenAlex v 6.501 [62] using SNPs with a call-rate ≥ 75% (5531 SNPs).

### Patterns of genetic differentiation along the genome

The *F*_*ST*_ for each window of the genome [63], Tajima’s D [64], diversity from Nei (*π*, average pairwise differences among individuals chosen randomly from the sample population) [65], nucleotide diversity within the population [66, 67] and Watterson’s θ (θ_W_, estimation of population mutation rate calculated on the basis of the number of segregating sites) [68], were estimated using non-overlapping 100 Kb sliding windows in PopGenome package for R [57, 69]. The patterns of variation across the gene pools, as well as, between Cultivars/Lines and Landraces within each gene pool were calculated. The ggplot2 R package (http://ggplot2.org/) was used to create the graphs for patterns of variation [70].

### SNPs under signature of selection (outliers)

The outlier SNPs were detected using two methods: 1) Method proposed by Foll and Gaggiotti [71] implemented in the BayeScan 2.0, which estimates the probability of each locus to be under selection using MCMC. The analysis was performed using 20 pilot runs with 5000 interactions, burn-in of 100,000 followed by 100,000 interactions (“thinning interval” equal to 20 and sample size of 5000), with a probability > 1. The analysis was performed three times to ensure robustness and only the outliers loci identified across all the runs were considered. 2) Hierarchical method of Excoffier et al. [72] implemented in Arlequin v 3.5.2.2 [73], which identified outlier loci by comparing the levels of genetic diversity and differentiation among populations. The hierarchical island model was simulated with two groups (Andean and Mesoamerican), two demes per group with 20,000 simulations to generate an *F*_*ST*_ joint distribution versus heterozygosity. Those loci that fall outside the 95% confidence interval were considered outliers.

### Linkage disequilibrium (LD) and haplotype blocks

LD was estimated using SNPs with MAF ≥ 0.05 and the pairwise LD measures were calculated by the usual method (r^2^) and corrected for bias due to population structure (K = 2) and relatedness (r^2^_SV_) using the LDcorSV package for R [57, 74]. The Genetic Relationship Matrix (GRM) was estimated using the algorithm proposed by Yang et al. [75] using GCTA software [76]. LD decay (half of the maximum value) was explained by the nonlinear model proposed by Hill and Weir [77] and adjusted to the nls function in R [57]. Haplotypic blocks were identified using Haploview 4.2 [78] based on the confidence interval method described by Gabriel et al. [79]: MAF ≥ 0.05 and call-rate ≥ 75%. Heterozygous loci were considered missing data.

### Genetic diversity distribution based on temperature and rainfall maps

#### Genetic diversity of landraces heatmap

Spatial analysis of genetic diversity (*H*_*E*_) was performed applying an individual-centered approach as described by [80] and adapted from the Wombling method [81]. *H*_*E*_ estimates were obtained using a hierarchical procedure, with a 150 km neighborhood grid used to avoid spatial autocorrelation between groups. In cases in which only one accession was represented in a given region, *H*_*E*_ represents diversity only for this accession. This analysis was performed using the “sHe” function of the R package “biotools” [57, 82].

#### Georeferencing landraces in thematic maps of climate in Brazil

The Brazilian maps were derived from the Brazilian Institute of Geography and Statistics (IBGE, Department of Cartography, 2016). Data from rainfall and climate/temperature were obtained from the Institute of Forest Research and Studies (IPEF). The software ArcGIS, based on Geographic Information System (SIG), was used to define areas on the maps. Landraces were geographically placed on the maps using the associated coordinate information.

## Results

### Genotyping using DArTseq

The 188 beans analyzed by DArTseq comprised a mini core group derived from the Brazilian common bean core collection (600 accessions) and are representative of the most genetically diverse accessions identified by microsatellite markers analysis (data not shown). For the SNP markers generated in DArTseq (Additional file 2), robust parameters were implemented: (1) call-rate ranging from 0.50 to 1.00, with an average of 92%, in other words, only ~8% missing data for each marker; and (2) high scoring reproducibility, ranging from 96.85 to 100%. The averages of homozygotes and heterozygotes were 0.88 and 0.04, respectively. Polymorphism content (PIC) ranged from 0.23 to 0.5, with an average of 0.44, and the minor-allele frequency (MAF) ranged from 0.13 to 0.5, with an average of 0.35. A total of 6286 SNPs were obtained from 181 accessions, of which only seven genotypes (3.72%) failed to generate sequence information.

### Structural and functional characterization of SNPs

**Fig. 1:**
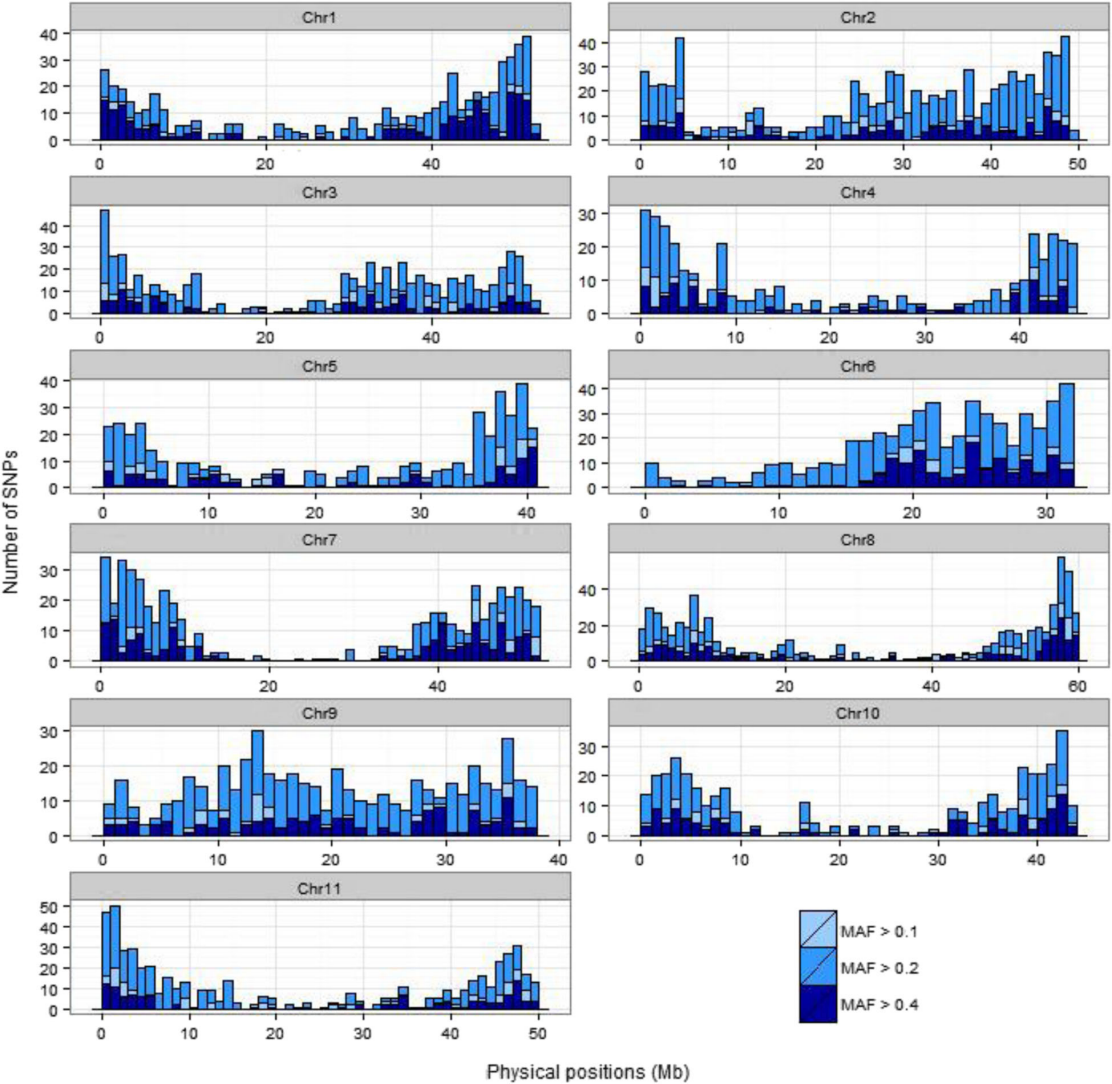
Distribution and positioning of SNPs along *P. vulgaris* chromosomes

From the 6286 SNP flanking regions, 308 were anchored to the same genomic position, 5961 (94.82%) showed alignment in the genome, of which 5311 (89.09%) aligned to a single region and 650 (10.90%) presented multiple alignments (ranging from two to 88). The sequences aligned to the 11 chromosomes and 12 scaffolds. The average number of SNPs per chromosome was 541, ranging from 389 on chromosome 4 to 792 on chromosome 2 (Table 1, Fig. 1). Based on Phytozome database, 15 SNPs aligned with 12 scaffolds and 325 SNPs did not align with the genome. An average of one SNP every 86,503 base pairs was estimated. Regarding the polymorphism types, transition (Ts) was the most abundant (3299 events, 55.30%), being most frequently cytosine to thymine (923), followed by transversions (Tv) with 2655 events (44.70%). The ratio of Ts/Tv was 1.24. A total of SNPs in genes was 43.3%, of which 20.8% in exons, 17.3% in introns, 4% in UTR region and 1.2% in splicing sites.

**Table 1:** SNPs-DArTseq distribution by common bean chromosomes

Chromosome	Number of SNPs	Chromosome size (kbp)^a^	Mean of SNP per Mbp
1	533	52183.50	10.21
2	792	49033.70	16.15
3	623	52218.60	11.93
4	389	45793.20	8.49
5	431	40237.50	10.71
6	532	31973.20	16.64
7	537	51698.40	10.39
8	656	59634.60	11.00
9	523	37399.60	13.98
10	401	43213.20	9.28
11	529	50203.60	10.54
Scaffolds	15	-	-
Total	5961	513589.10	11.58

^a^Schmutz et al. [9]

**Fig. 2:**
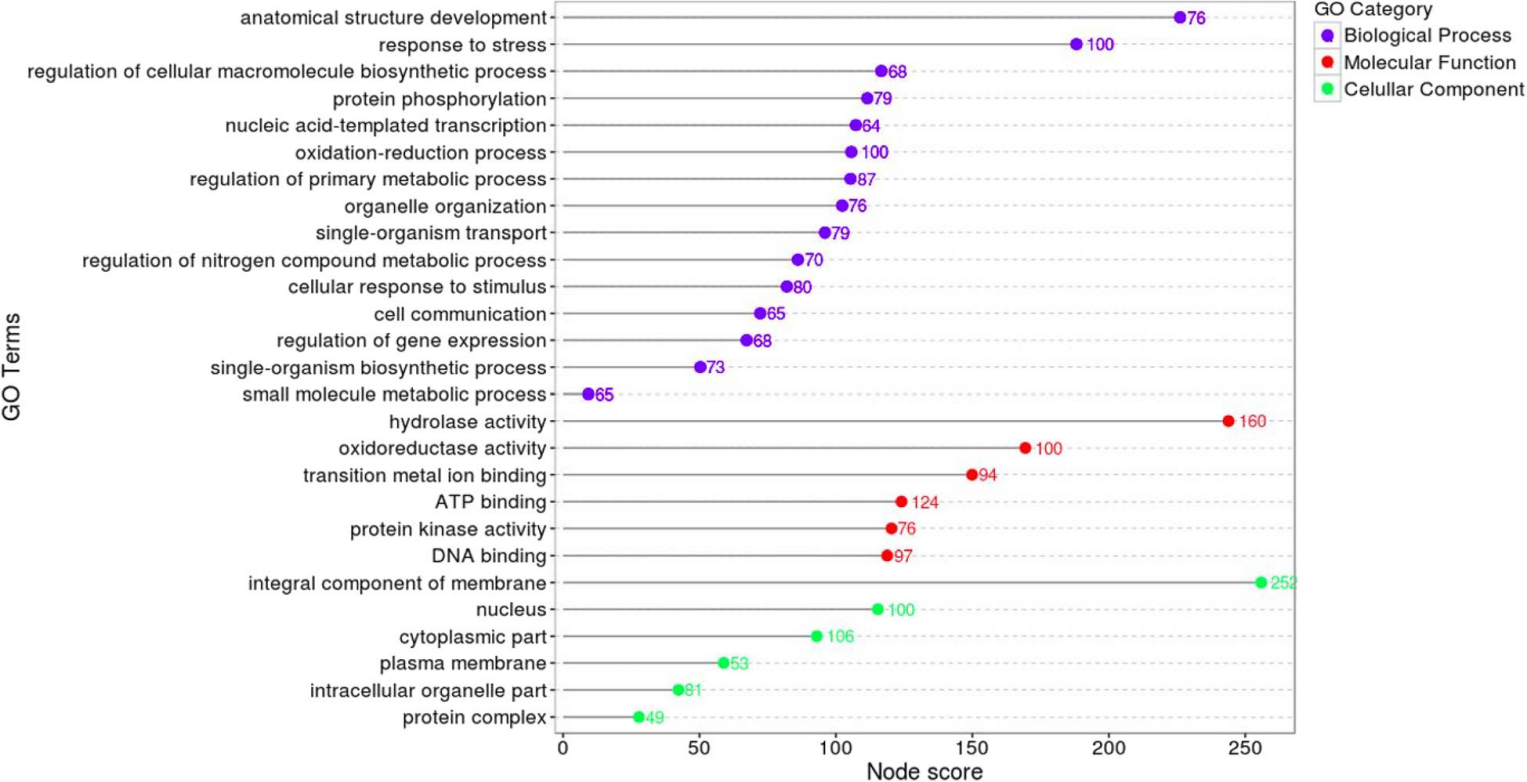
Functional annotation of SNPs with high and moderate impact predicted by SnpEff. The terms were filtered according to the node score. The numbers represent the amount of transcripts related to each term

A total of 12,217 functional effects for SNP variants were predicted for 5,954 SNPs, providing information on the location of all isoforms, genic, and intergenic regions. The predicted effects were of modifier type (77.8%), low impact (14.22%), moderate impact (7.92%), and high impact (0.05%). Most SNPs with predicted effects were observed in genic regions (6950), of which 20.82% and 17.30% were observed within exons and introns, respectively, with the remaining in non-translated regions. In genic flanking sequences (5 kb window) 5,267 effects were identified, of which 58.21% and 41.79% occurred in downstream and upstream regions, respectively. SNP effects categorized as moderate and high, were identified in 901 transcripts, of which 810 were mapped and 777 were fully annotated (Additional file 3). These genes were related to a variety of mechanisms, such as plant development and multiple stress response pathways (Fig. 2). Among the 777 annotated transcripts, 359 were identified as enzymes, mainly transferases (129) and hydrolases (125; Additional file 4). Genes involved in metabolic pathways are described in Additional file 5.

For the six genes categorized as highly impacted, their gene annotations were proposed to show the SNP position relative to the gene introns and exons (Additional file 6). For three of the genes, the high impact SNPs affected the alternative splicing, while for the remaining genes, the SNP allele change generated a stop codon. In the Mesoamerican gene pool, four genes were predominantly homozygous (≥95.5%) for the non-disruptive (favorable) SNP allele, while the genes Phvul.006G191000 (a splicing factor) and Phvul.010G1404000 (ABC transporter) were mostly homozygous for the favorable SNP allele in the Andean (≥92.1%). Two genes (Phvul.006G023300; Phvul.003G030200) had an increased frequency of the genotypes that is homozygous for the favorable SNP alleles in both gene pools. Only one gene (Phvul.010G1404000) in the Mesoamerican gene pool showed a homozygous favorable allele (32.4%) and unfavorable allele (57.7%).

### Germplasm genetic structure

**Fig. 3:**
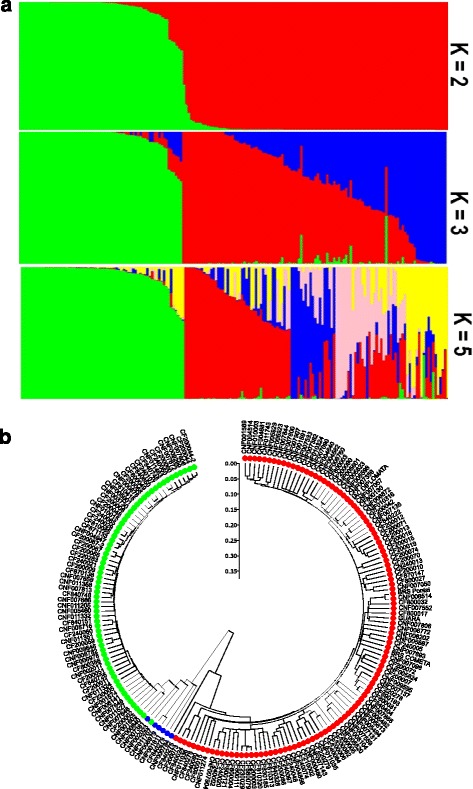
Population structure **a** Population structure inferred by the Bayesian approach based on SNPs for K = 2 to 5. K = 2 subdivided genotypes in Mesoamerican (*red*) and Andean (*green*). K = 3 subdivided the Mesoamerican genotypes into two groups: M1 (*red*) and M2 (*blue*). K = 5 subdivided the Mesoamerican genotypes into four groups: M1 (*red*), M2 (*blue*), M3 (*pink*), and M4 (*yellow*). **b** Dendrogram showing the division between the two gene pools: Andean (*green*), Mesoamerican (*red*), and admixture (*blue*)

Population structure analysis performed using 580 SNPs in LE revealed K = 2 as the most likely, with the subdivision in Andean (64) and Mesoamerican (111) gene pools and six genotypes (3.87%) as admixture (Fig. 3a). Five of the genotypes with admixture (ranging from 62 to 69%) were mainly from Andean origin: four cultivars/lines developed by international institutions and one Brazilian landrace from Rio Grande do Sul state (white or brindle grains). The genotype with a predominance of Mesoamerican germplasm (~65%) is a cultivar/line with brindle grain type from Russia (CNF000784). For K = 3, the Mesoamerican groups were fragmented in two (M1 and M2) in addition to 45 genotypes with admixture. The M1 group was composed by 46 accessions (q ≥ 0.7) of which 74% (34) were black grain types from Brazilian and international cultivars/lines. M2 contained 20 Brazilian genotypes (q ≥ 0.7), 17 landraces and three cultivars/lines, without grain type prevalence. For K = 5, an additional fragmentation within the Mesoamerican gene pool was observed (M1, M2, M3, and M4). M1 was formed by 28 genotypes, 20 cultivars/lines and eight landraces, with predominance (82.14%) of the black grain type. M2 contained seven accessions from Brazil (six landraces and one cultivar/line), of which 43% were of the yellow grain type. The M3 group was represented by six Brazilian genotypes (four landraces and two cultivars/lines) with a carioca commercial grain type. Finally, M4, with eight genotypes (six landraces and three cultivars/lines), had different types of grain (62.5% of brown and red type).

**Fig. 4:**
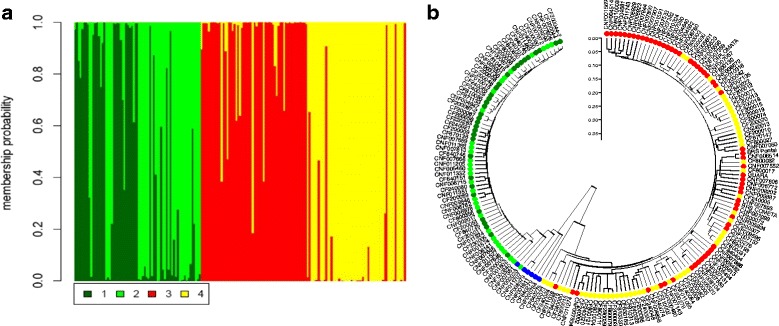
**a** DAPC using the set of 5531 SNPs showing the separation between Mesoamerican cultivars/lines (*red*) and landraces (*yellow*). 1: Andean cultivars/lines; 2: Andean landraces; 3: Mesoamerican cultivars/lines; 4: Mesoamerican landraces; **b** Dendrogram showing the division between the two gene pools: Andean cultivars/lines (*dark green*), Andean landraces (*light green*), admixture (*blue*), Mesoamerican cultivars/lines (*red*), and Mesoamerican landraces (*yellow*)

The tools implemented in DAPC revealed a more complex population structure in the Mesoamerican by landraces and lines/cultivars (Fig. 4). The dendrogram shows the same division found in Structure (K = 2; Fig. 3b).

### Analysis of population genetics structure

The analysis of genetic diversity revealed a total of 5531 polymorphic SNPs, of which approximately 26% distinguished the Andean from the Mesoamerican genepools. In general, Mesoamerican germplasm presented an increased number of polymorphic loci and private alleles, as well as higher mean gene diversity and reduced observed heterozygosity (Table 2). There is considerable inbreeding (F) within each gene pool (values of 0.561 and 0.652), but there is also a strong contribution of the subdivision for total inbreeding (F = 0.90), which is evidenced by the high *F*_*ST*_ value between groups (0.747). The combined set of markers generated an overall exclusion power of 100%, whereas 28 SNPs distinguished all genotypes (Table 2).

**Table 2:** Genetic diversity and divergence within Andean and Mesoamerican gene pools

Group	S	P	NAP	H_O_ (SE)	H_E_ (SE)	F (SE)	F_ST_ (SE)	F_IS_ (SE)	F_IT_ (SE)	PI	PE
Andean	64	82.99%	511	0.040 ± 0.002	0.102 ± 0.002	0.561 ± 0.006	0.747 ± 0.001	0.822 ± 0.001	0.955 ± 0.0031	1.05E-249	1
Mesoamerican	111	90.76%	941	0.035 ± 0.001	0.168 ± 0.002	0.652 ± 0.006				0	1
Total	175	100	-	0.0373 ± 0.001	0.4425 ± 0.001	0.9082 ± 0.003				0	1

The sample size (*S*), percentage of polymorphic loci (*P*), number of private alleles (NAP), observed heterozygosity (*H*_*O*_), gene diversity (*H*_E_), inbreeding coefficient (*F*), genetic differentiation (*F*_*ST*_), fixation index (*F*_*IS*_), total inbreeding (*F*_*IT*_), probability of identity (PI), probability of exclusion (PE), and standard deviations (SE) are presented

High numbers of polymorphic SNPs were identified for Mesoamerican (87.51%) and Andean (88.39%) cultivars/lines compared to the landraces (Mesoamerican = 90.78%. Andean = 73.49%). The *H*_*E*_ values for the Mesoamerican group were 0.177 and 0.185 for cultivars/lines and landraces, respectively, while for the Andean, the corresponding values were 0.145 and 0.099. In both gene pools, the estimates of *F*_*ST*_ between cultivars/lines and landraces were 0.031 (*p* >0.001) and 0.012 (*p* >0.002) for Mesoamerican and Andean, respectively. Within the Andean gene pool, cultivars/lines presented 1,217 private alleles, while in landraces it was 533 (Table 3).

**Table 3:** Genetic diversity and divergence among cultivars/lines and landraces of the Andean and Mesoamerican gene pools

Group		S	P	NAP	H_O_ (SE)	H_E_ (SE)	F (SE)	F_ST_ (SE)	F_IS_ (SE)	F_IT_ (SE)
Mesoamerican	Cult/Lines^a^	57	87.51%	463	0.038 ± 0.001	0.177 ± 0.003	0.652 ± 0.006	0.031 ± 0.001	0.836 ± 0.001	0.841 ± 0.001
	Landraces	54	90.78%	627	0.040 ± 0.001	0.185 ± 0.002	0.646 ± 0.006			
	Total	111	100.00%	-	0.039 ± 0.001	0.185 ± 0.002	0.652 ± 0.006			
Andean	Cult/Lines^a^	31	88.39%	1217	0.046 ± 0.002	0.145 ± 0.002	0.647 ± 0.007	0.012 ± 0.002	0.738 ± 0.001	0.741 ± 0.001
	Landraces	33	73.49%	533	0.050 ± 0.002	0.099 ± 0.002	0.377 ± 0.007			
	Total	64	100.00%	-	0.048 ± 0.002	0.123 ± 0.002	0.561 ± 0.007			

The sample size (*S*), percentage of polymorphic loci (*P*), number of private alleles (NAP), observed heterozygosity (*H*_*O*_), gene diversity (*H*_E_), inbreeding coefficient (*F*), genetic differentiation (*F*_*ST*_), fixation index (*F*_*IS*_), total inbreeding (*F*_*IT*_), and standard deviations (SE) are presented

^a^Cult/Lines: cultivars/lines

### Patterns of genetic differentiation along the genome

**Fig. 5:**
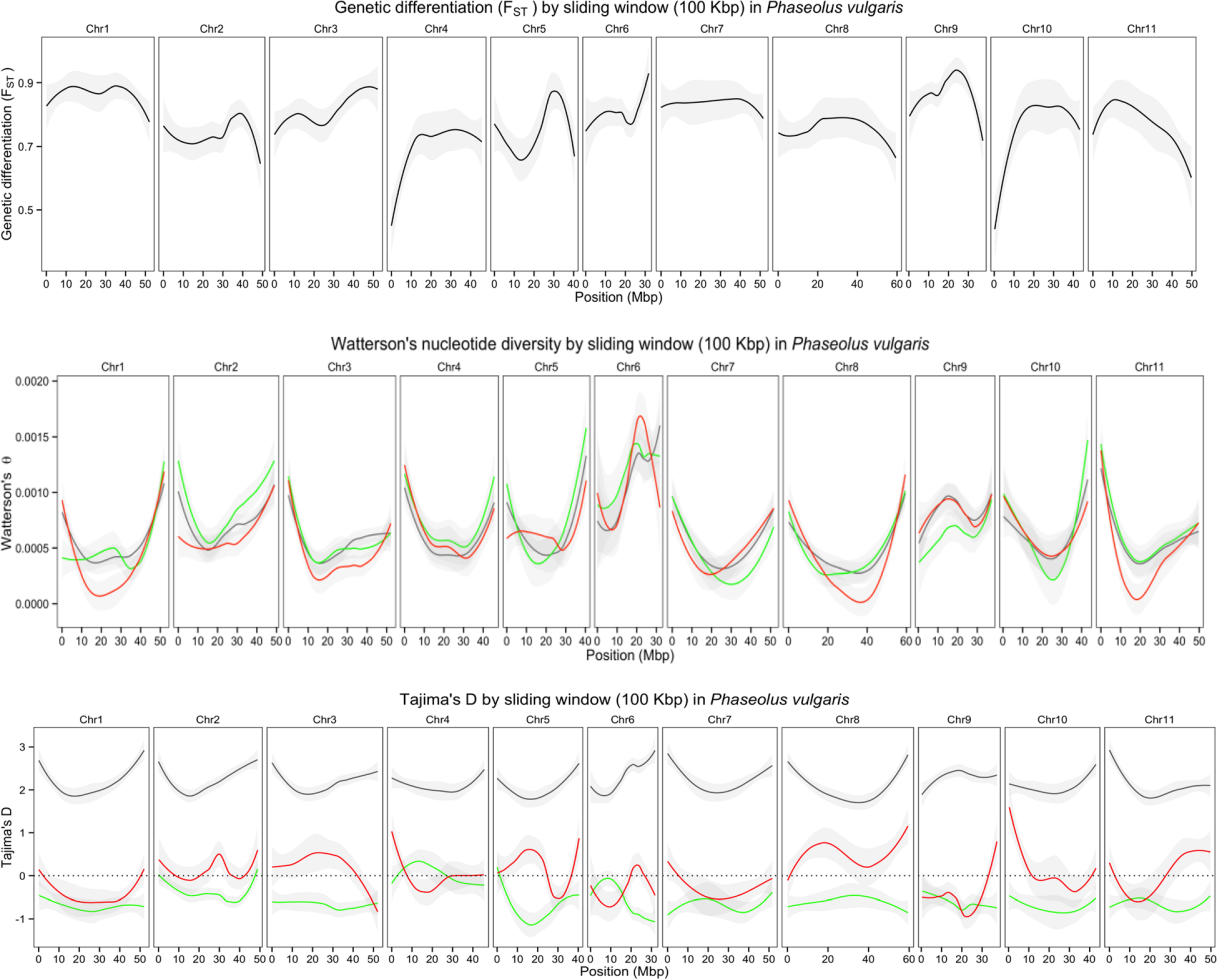
Genome-wide loess curves for genetic differentiation (*F*_ST_), Watterson’s θ (θ_W_), and Tajima’s D for all 11 chromosomes in the *P. vulgaris* genome for each gene pool. *F*_ST_ is given as an average across all pairwise comparisons between Andean and Mesoamerican gene pools. The results of Tajima’s D and θ_W_ are given for each gene pool separately: Mesoamerican (*red*) and Andean (*green*), and estimated for the whole population (*grey*). *F*_ST_, Tajima’s D and θ_W_ summary statistics were calculated for each 100 kb non-overlapping sliding window

*F*_ST_ was high between the gene pools for the majority of the chromosomes, with the highest level at chromosomes 1 and 9 (Fig. 5a). The overall differentiation among cultivars/lines and landraces was lower for the Andean germplasm (*F*_ST_ = 0.0082) compared to the Mesoamerican (*F*_ST_ = 0.0218; Additional file 7). The average value of *π* over the whole population, based on 5,241 SNPs, was 0.0171 (±0.001) and was greatly reduced for the Andean (*π* = 0.0017 ± 0.0002, 3,889 SNPs) and Mesoamerican (*π* = 0.0045 ± 0.0006; 3,957 SNPs) groups (Table 4). These values were consistent with an MAF > 0.3 for 4,210 (80%) SNPs in the whole population and an MAF < 0.1 for about half of SNPs into the Andean and Mesoamerican groups. Considering the germplasm stratum, the *π* value was 0.0044 (±0.0005) for the cultivars/lines and 0.0043 for the landraces of Mesoamerican origin, with a similar distribution of SNPs into MAF classes. Reduced values were observed for the cultivars/lines (0.0022) and landraces (0.0013) of Andean origin, probably due to the additional set of SNPs with MAF > 0.1 and ≤ 0.2 (Additional file 8).

**Table 4:** Summary of the *P. vulgaris* genome-wide diversity based on SNPs-DArTseq

Gene pool	Group	N	S	Θw	SE – θw	NDw	SE - NDw	π	SE - π	P	M
Andean	Cult/Lines^a^	31	3506	0.000777	0.000015	0.000554	0.000014	0.002171	0.000281	3858	1415
	Landraces	33	2647	0.000580	0.000013	0.000386	0.000011	0.001357	0.000228	3190	2083
	Total	64	3889	0.000728	0.000013	0.000471	0.000012	0.001781	0.000266	4364	909
Mesoamerican	Cult/Lines^a^	57	3283	0.000641	0.000014	0.000665	0.000020	0.004386	0.000488	4177	1096
	Landraces	54	3460	0.000667	0.000014	0.000677	0.000019	0.004330	0.000566	4340	933
	Total	111	3957	0.000667	0.000012	0.000685	0.000019	0.004541	0.000572	4778	495
Whole	All	181	5241	0.000713	0.000010	0.002038	0.000029	0.017125	0.001540	5273	0

The number of samples (N), number of segregating sites (S), Watterson’s nucleotide diversity (θw), nucleotide diversity within (NDw), diversity from Nei (*π*), number of polymorphic SNPs (P), number of monomorphic SNPs (M), and standard deviations (SE) are presented

^a^Cult/Lines: cultivars/lines

The Watterson’s Mean θ (θ_W_) for all individuals was 0.00071 (±0.00001), with a lower value estimated for the 33 Andean landraces (0.00058). The θ_W_, Tajima’s D, and *F*_ST_ estimates were highly variable across the *P. vulgaris* genome (Fig. 5 and Additional file 7) and regions that displayed high values of *F*_ST_ also presented elevated LD (data not shown) and reduced θ_W_ and Tajima’s D (Fig. 5), mostly in centromeric regions. For the Andean accessions, negative Tajima’s D values were observed for all chromosomes except for chromosome 4, which could indicate that positive selection in the Andean group is driving divergence between the gene pools, as evidenced by the correlation of centromeres and regions of elevated *F*_ST_ (Fig. 5a and c). For the Mesoamerican group, Tajima’s D values were variable across the genome and some regions, such as in the chromosome 5 approximately 10-20 Mbp, presented high Tajima’s D values and low *F*_ST_, indicating balancing selection (Fig. 5a and c). Conversely, in the same chromosome 5, a region near 30 Mbp had a low Tajima’s D value and high *F*_ST_, indicating possible positive selection (Fig. 5a and C).

### Loci under signature selection (outliers)

A total of 16 and 59 outlier SNPs were identified based on BayeScan (q < 0.05) and Arlequin (p < 0.05), respectively, of which 16 loci were common to both analyses. From the 59 SNPs, 54 aligned over the 11 chromosomes (with the highest abundance on chromosomes 1 and 9), with an average of one SNP every 8.6 million bases. Across the genome, ~41% of SNPs were identified within genes (17.27% in introns, 20.91% in exons, and 2.73% in the 5’ UTR), while the remaining (~59%) were in intergenic regions. The analysis of SNP effect on the outliers revealed a total of 110 effects predicted for 54 outliers, of which 11% were low-impact, 10% moderate and 79.09% modifier type. We identified 91 transcripts affected by 54 SNPs under selection, of which 82 presented homology to the non-redundant (nr) protein database and 71 were annotated (Additional file 9). Based on GO, within the categories of “cellular component,” “biological process,” and “molecular function,” most genes were assigned to “integral component of membrane, plasma membrane, and cytoplasmic part,” “DNA binding, ATP binding, and ligase activity,” and “DNA metabolic process, transmembrane transport, and signal transduction,” respectively (Additional file 10). In addition, 45 SNP outliers were identified in metabolic pathways (Additional file 11).

Within the Mesoamerican gene pools (comparing between landraces and cultivars/lines), 15 outlier SNPs common to both analyses were identified distributed around chromosomes 2, 7, 8, and 9. 131 transcripts were affected by these SNPs, and 116 of these have been annotated (Additional file 12). For the Andean group, a set of 18 outlier SNPs, mainly in chromosome 10, were associated with 42 transcripts, of which 35 were annotated (Additional file 13). Only one outlier loci (3381974_16_T_C) was common to both gene pools. The most abundant functional terms within one of the three GO categories is described in Additional file 14.

### LD decay

**Fig. 6:**
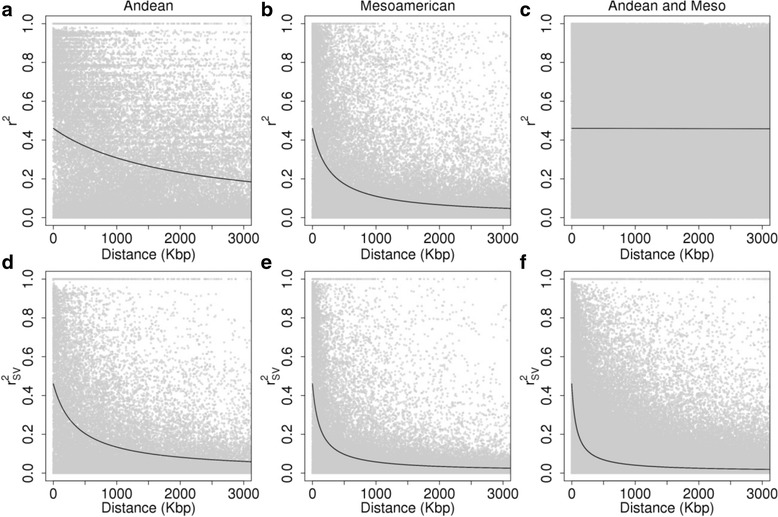
LD decay without correction (r^2^, panels **a**, **b** and **c**) and corrected for relatedness and population structure (r^2^_sv,_ panels **d**, **e** and **f**) for the Mesoamerican (**a** and **d**), Andean (**b** and **e**) and the grouped accessions (**c** and **f**)

The LD decay in the Andean gene pool (Fig. 6a and d) was slower than in the Mesoamerican gene pool (Fig. 6b and e). For Andean group, LD with correction for relatedness and structure showed a decay dropped to half (*r* ^2^ = 0.23) at a distance of ~2055 Kb and ~395 Kb for r^2^ and r^2^_SV_, respectively, while for the Mesoamerican, half decay was observed at distances of ~312 Kb and ~130 Kb. For accessions overall without correction (*r* ^2^), no decay was observed within the 3000 Kb window (Fig. 6c), while with correction (r^2^_SV_) LD decreased to half at 88 Kb (Fig. 6f). Within the landraces, the r^2^ was estimated to be 1722 Kb and 389 Kb, for the Andean and Mesoamerican groups, respectively, and for the stratum cultivars/lines, it was 4040 Kb and 428 Kb.

Through haplotype analysis, a set of 437 blocks representative of the 11 chromosomes, ranging from 31 (chromosome 1) to 62 (chromosome 8) were identified. A total of 4354 SNPs (82.57%) were distributed in these blocks, with an average of ~10 SNPs per block. Chromosomes 9 (90.12%) and 4 (71.77%) had the highest and lowest percentage of SNPs within blocks, respectively. The average block size was 842.2 Kb, and the largest block was in chromosome 3, with 8634 Kb and 21 SNPs. The maximum and minimum frequency of haplotypes was 0.850 and 0.010, respectively, with the most common haplotype located on chromosome 7. On average, 71.66% of the genome was covered by the blocks (Table 5). A larger number of blocks were identified in the Mesoamerican group (248 blocks), compared to the Andean group (98 blocks), comprising 798 (3.18 SNPs/block) and 592 (6.0 SNPs/block) SNPs, respectively. In both gene pools, chromosome 2 presented the highest number of blocks (25 for Andean and 41 for Mesoamerican groups, Table 5).

**Table 5:** Haplotype blocks identification using the SNPs-DArTseq for the all accessions and within Andean (AND) and Mesoamerican (MESO) gene pools

Chromosomes	Total of blocks			Average SNP/block			Blocks size (kb)			Physical length (kb)^a^	Block coverage (%)
	All	AND	MESO	All	AND	MESO	All	AND	MESO	All	All
1	31	2	22	13.94	4	3.64	42497.69	67.1	1831.76	52183.5	81.44
2	56	25	41	10.71	8.16	1.07	38734.17	22616.37	10256.22	49033.7	78.99
3	37	5	20	12.95	2.2	4.2	42067.03	28.27	6336.05	52218.6	80.56
4	35	19	18	6.83	6.11	5.22	30826.24	18181.43	20469.35	45793.2	67.32
5	35	9	20	8.89	7.33	3.8	28497.35	3365.23	10704.77	40237.5	70.82
6	40	10	23	10.58	7.4	3	24709.16	132885.33	1542.29	31973.2	77.28
7	37	5	18	11.35	2	2.78	35632.69	31.9	506.8	51698.4	68.92
8	62	5	36	6.9	3.8	3.5	35786.86	354.41	2954.98	59634.6	60.01
9	33	4	16	13.27	4.5	3.63	30338.75	1362.9	1922.26	37399.6	81.12
10	36	6	17	6.89	5.5	3.06	26847.85	1712.86	3235.07	43213.2	62.13
11	35	8	17	9.6	4.13	3.29	32104.09	659.16	2795.36	50203.6	63.95
Total	437	98	248	9.96	6.04	3.18	368041.88	181264.96	62554.91	513589.1	71.66^b^

^a^Schmutz et al. [9]

^b^Average genome block coverage for the all accessions

### Genetic analysis based on a low*-*density SNP panel

A total of 560 SNPs were selected for the assessment of genetic diversity in common bean (Additional file 15). These SNPs were polymorphic in both gene pools, with MAF > 0.05, r^2^ < 0.5, an average *H*_*E*_ = 0.401 and were distributed over the 11 chromosomes. The *F*-values between the Andean and Mesoamerican groups were *F*_ST_ = 0.411 (±0.001), *F*_IS_ = 0.826 (±0.001), and *F*_IT_ = 0.897 (±0.001). DAPC revealed a structure similar to those obtained for the whole set of SNPs (5531) (Additional file 16). Within the Andean gene pool, 88.57% and 72.50% of SNPs were polymorphic for the landraces and cultivars/lines, respectively, with *F*_ST_ estimated at 0.010 ± 0.001. For the Mesoamerican accessions, ~97% of SNPs were polymorphic in both stratum, with an estimated *F*_ST_ of 0.034 ± 0.001.

### Genetic diversity distribution based on temperature and rainfall maps

**Fig. 7:**
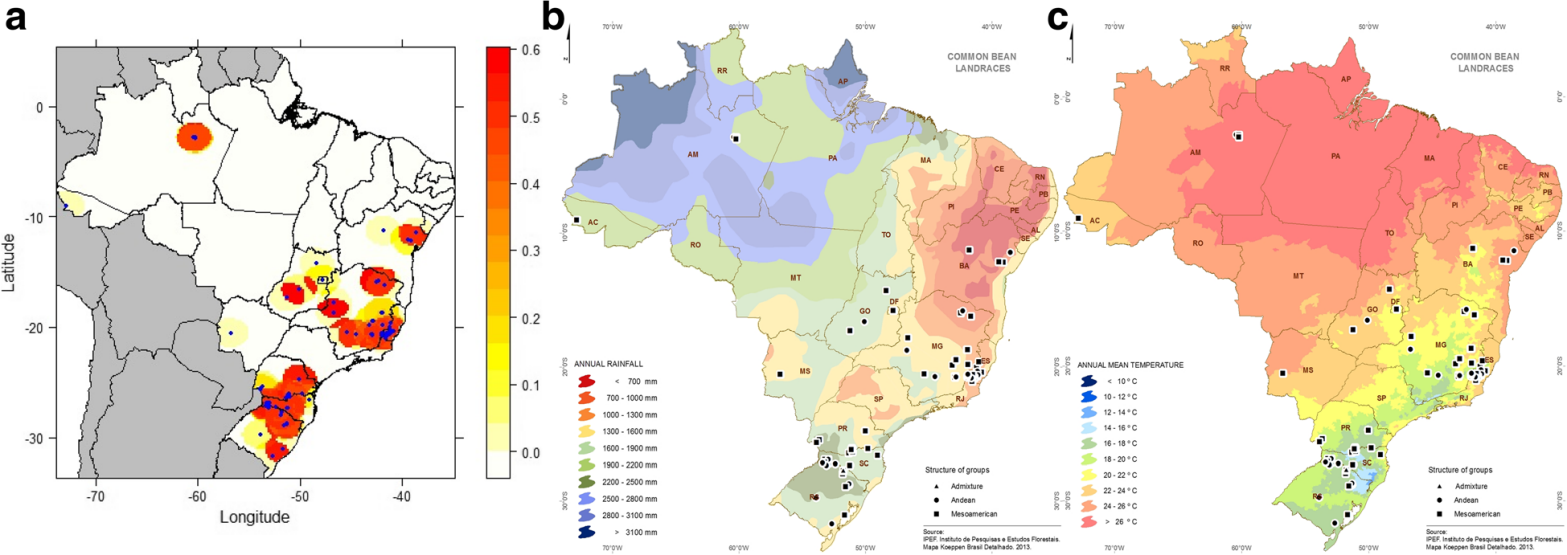
Spatial distribution of the genetic diversity (**a**) on the maps of total annual rainfall (**b**) and average annual temperature (**c**). The *circle* represents accessions from Mesoamerican origin and *square* from Andean origin. Source: IBGE, 2016 with modifications

The highest estimates of *H*_*E*_ were observed in areas containing germplasm of Andean and Mesoamerican origin, as well as accessions characterized as admixtures by structure analysis (Fig. 7). Within gene pools, the average genetic distance and *H*_*E*_ were estimated at 0.1414 and 0.185 for the Mesoamerican, respectively, and 0.054 and 0.099 for the Andean gene pools, respectively. However, the highest *H*_*E*_ of a set of four accessions from regions with extreme temperature (three from regions with ≥ 26 °C - CF200005, CF200003, CF200002 - and one from regions ranging from 14 to 16 °C - CF200070) was 0.411. For the regions with low precipitation (≤700 to 1000 mm), the *H*_*E*_ of the seven accessions (CF810313, CF810320, CF840404, CF810415, CF800027, CF800032, CF800049) was 0.419.

## Discussion

### Genotyping with DArTseq

The SNP markers derived using DArTseq demonstrated that this technology is an efficient method of genotyping with broad genome coverage and can be useful for analyses of genetic diversity in a common bean germplasm pool composed of landraces and cultivars/lines. The sequencing of two important varieties of common bean representative of the Andean and Mesoamerican groups [9, 18] has allowed the identification and determination of genomic positions of SNPs with several scientific implications. Among the 6286 SNPs identified, 94.82% were placed on the *P. vulgaris* genome, supporting the analysis of population structure, LD, and identification of genomic regions under selection that have an impact on crop improvement research. The average call-rate was 92%, close to the value of 91.3% previously reported for watermelon [40], and the scoring reproducibility of 99.44% was consistent with the value described for wheat (98.5%) [83]. A high density of SNPs was obtained (SNP/86Kbp) compared to our previous report (SNP/500Kbp), which was based on RADseq technology [31], increasing the genome resolution for subsequent analyses. The combination of restriction enzymes used in DArTseq (*Pstl-Msel*) resulted in the more frequent appearance of SNPs, as reported by Schröder et al. [36]. Within the Andean (83%) and Mesoamerican (91%) gene pools, a larger proportion of polymorphisms were identified, which was higher than previously reported using SNPs-RAD (Andean: 72.7% and Mesoamerican 83.3%) [31]. The considerable level of SNP polymorphism within gene pools, in addition to their wide genomic representativeness over the genome (99.78%), is favorable to reduce the ascertainment bias given a more uniform and realistic distribution of allelic frequency over the whole population.

### Genetic diversity

DArTseq also allowed the detection of SNPs with high diversity (*n* = 181, *H*_*E*_ = 0.442), compared to the SNPs identified by RAD (*n* = 95, *H*_*E*_ = 0.384), Valdisser et al. [31] and SNPs identified between BAT93 and Jalo EPP558 (*n* = 88, *H*_*E*_ = 0.390), Müller et al. [29]. For the Mesoamerican, genetic diversity (*H*_*E*_ = 0.168; *n* = 111) was close to values obtained by Rodriguez et al. [16] for domesticated bean (*H*_*E*_ = 0.157; *n* = 100); however, lower compared to the studies of Cichy et al. [33] (*H*_*E*_ = 0.233; *n* = 21), who characterized a more diverse set of accessions that are from different geographic origin, breeding program, grain type, and growth habit. In our Mesoamerican germplasm, the only structure observed was by grain type (K = 4) [29, 31], with a high proportion of admixture (55.85%) resulted from long-term genetic improvement and relationships among breeding germplasm. The patterns of nucleotide diversity along the whole genome also revealed a reduction in the order of 60.8% for the Andean (*π* = 0.001781), compared to the Mesoamerican (*π* = 0.004541) germplasm. The lowest diversity in the Andean is expected (*H*_*E*_ = 0.102; *n* = 64; *p* 0.05) due to the prevalence of Mesoamerican introduction and domestication in Brazil [10, 22], in addition to global historical events of domestication in common bean evolution [9, 84, 85]. The inter-gene pool hybridization had a positive impact on Andean diversity [9, 86], whereas the Mesoamerican group was exposed to more frequent events of recombination on a broader genetic base, generating more diversity [87, 88].

In the present study, all landraces originated from Brazil and only the breeding germplasm included introductions. From the 87 Brazilian landraces, lower estimates of *H*_*E*_ and *π* were reported for the Andean, compared to the Mesoamerican. Moreover, the diversity of the Andean Brazilian landraces (*n* = 33, *H*_*E*_ = 0.099) reduces only in the order of 1.3 x compared to the diversity estimated by Cichy et al. [33] which analyzed accessions representative of ~30 countries using the BARCBean6K_3 bead chip (n = 201 landraces; *H*_*E*_ = 0.128). Therefore, there is a strong indication that Brazil may be a center of secondary domestication with high diversity that deserves a further characterization of the remaining accessions and integrates into the Brazilian core collection. For the Andean cultivars/lines (*n* = 31), even represented by accessions of 22 countries, the *π*_*C/L*_ (0.002171) was almost half the value for the Mesoamerican (*n* = 57; *π*_*C/L*_ = 0.004389) used predominantly in breeding program for improving agronomic traits [22].

Regarding the spatial distribution of the genetic diversity, no significant relationship between genetic and physical distances was identified (data not shown). By overlapping the thematic and diversity maps, important sites to collect landraces, considering both genetic diversity and adaptability under hydric restriction and high temperature were identified. For breeding purposes, this is extremely valuable in terms of understanding adaptive responses and identification of target accessions genetically diverse with the potential to be integrated into bean pre-breeding programs, as well as germplasm resource management. Genetically diverse accessions originated from geographic areas with high temperature and reduced rainfall could be of potential use in common bean breeding programs in attempt to increase the frequency of favorable alleles, and consequently, increasing the potential to obtain inbred lines more tolerant to drought stress, which can be obtained, for example, through recurrent selection.

### SNP effect prediction

As expected, the SNP effects categorized as modifiers was more abundant and SNP with impact on protein efficiency and loss-of-function, that have a direct impact on gene function with adaptive interference during the course of selection, were reported in a smaller proportion [48]. Considering all loci under high impact, a low frequency of heterozygotes was observed, probably due to the autogamous nature of the common bean. From the six genes, two presented an increased proportion of homozygous for the favorable allele (Phvul.006G023300; Phvul.010G1404000, Phvul.003G030200) for both gene pools. This is an evidence that the disruptive allelic variant caused an impact that has been selected against during the process of domestication. These two genes are related to important cellular process, such as redox system essential in maintaining cellular homeostasis (feruloyl ortho-hydroxylase - Phvul.006G023300) and endonuclease implicated in RNA and ssDNA degradation involved in cell death (endonuclease 2-like - Phvul.003G030200). In general, we observed allelic frequency difference within each locus between the Andean and Mesoamerican gene pools, suggesting that different forces (e.g. selection, founder effects) might be acting upon these loci. As expected, for the Mesoamerican genotypes the frequencies of the favorable alleles was increased for four, of the six genes under high impact effect, in response to selective natural and artificial pressure. The analysis of SNPs with high impact provides important clues about the selective forces acting in germplasm adaptation. Alonso-Blanca et al. [89] reported a loss of gene function conferring an adaptive advantage under domestication for the processes of germination, dormancy, and flowering. In addition, during the process of domestication, loss of function can be considered an important factor for rapid evolution [90].

### Loci under selection

The process of domestication and artificial selection imposed by agriculture resulted in changes to allelic frequencies and allowed the identification of genomic regions under adaptive evolution using high-density SNP genotyping [9, 91, 92]. In this study, high-resolution genetic analysis and a diverse set of domesticated accessions adapted to specific environments and subjected to natural and artificial selection allowed the identification of SNPs potentially related to these adaptive processes. Genomic regions under selection were not homogeneous in the present study (predominant on chromosome 1, *F*_*ST*_ = 0.86 and chromosome 9, *F*_*ST*_ = 0.87), suggesting that distinct and broad genetic mechanisms were involved in the process of common bean domestication. Similar finds were reported by Schmutz et al. [9] who described a greater proportion of loci under selection on chromosomes 1, 2, and 10 in the Andean group, and on chromosomes 2, 7, and 9 in the Mesoamerican group. Among the genes under positive selection, we identified enrichment in terms related to cell membrane transporters, receptors, sensors, gene recombination/mutation, and the complex network of intra- and extracellular signaling that could be attributed to adaptive changes, providing the ability to respond earlier to abiotic or biotic stimuli. Tolerance to multiple stresses is expected since the plants suffer from several forms of stress during their life cycle, where a range of molecular mechanisms act together through complex pathways with important mechanisms of crosstalk among them [93]. Among the landrace and cultivar/line strata, a high number of outliers was reported in the accessions of Mesoamerican origin, which is consistent with the predominance of this germplasm in Brazil [10, 22] and, consequently, the higher selective pressure imposed on this germplasm. These genes are potential targets for plant breeders because of their roles in plant adaptation under variable environmental conditions. The understanding the effect of these genes on the phenotypes will have a positive impact on crop improvement [94].

Outlier SNPs associated with the same GO terms were reported in both gene pools. Among these, we highlighted integral components of membranes that could respond to plant demand to be more efficient in the process of water and nutrient transport, as well as the location of photoassimilates. Furthermore, several common transcripts related to the development of morpho-anatomical structures were reported, corroborating previous studies of QTLs involved in the domestication and diversification processes [95]. Selective pressure on these genes is expected because in the process of domestication, several traits were privileged, for example, the trend for increases in wheat grain mass, which is strongly associated with endosperm development [96] and growth habit, as a trait under strong selection in common bean domestication [97]. Selective pressure on genes related to the redox status, plant development, and response to biotic and abiotic stresses was preferably identified in the Mesoamerican group, while processes of protein phosphorylation and ATP-binding predominated in the Andean germplasm. These genes play a fundamental role in the stimuli and signal processing of multiple stress responses, which are fundamental to plant adaptation in the evolution and domestication [98]. In addition, transcription factors and other genes related to the regulation of gene expression were also under selection. Genes related to the same mechanisms and associated with QTLs controlling domestication-related traits were reported by Doebley et al. [99]. Similar of those observed in maize [100] and soybean [92], transcription factors are abundant among genes under selection acting to regulate several process, such as grown habit, flowering, grain size, dormancy, and others [101]. Lastly, genes related to secondary metabolites that are known to respond to plant interactions with environmental changes, such as drought, radiation intensity, and pest attacks [102], were also identified.

Our data showed that, several genes under selection in both pools were related to pathways of primary metabolism, such as sucrose, amino acids, lipids and starch metabolism. These pathways are source of energy and carbon that broadly affect a range of cellular mechanisms. Interestingly, 11 distinct putative genes under selection were identified in both pools, six Mesoamerican and five Andean genes, related to the same Purine and Thiamine metabolic pathway revealing different signatures of selection associated with the same processes. Products derived from the Thiamine biosynthesis play role as a cofactor in important metabolic pathways, such as glycolysis, Krebs cycle, nitrogen assimilation, and have been shown to have functions in response to abiotic and biotic stress in plants [103]. The Purine metabolism plays central role in the cell involving production of nucleotides, coenzymes, and signaling molecules. In addition, this pathway also has a fundamental role in the process of nitrogen fixation that occurs in beans, which form molecules that transport the nitrogen through xylem under nitrogen fixing conditions [104].

### Linkage disequilibrium

A high proportion of alleles at low frequencies were observed within the gene pools, whereas for the whole set of accessions, most SNPs were present at high frequencies (≥0.3), reflecting the presence of fixed loci for alternative alleles between the gene pools. Without correcting for relatedness and structure, the LD presented elevates estimates of r^2^, and after the correction, an increase in decay was observed, showing that the evolutionary and breeding history [7, 85] strongly affect the association among markers. The LD decay observed in common bean extended over several bp (up to 88 Kb), compared to allogamous species, such as the Eucalyptus [105] and loblolly pine [106]. This was expected due to the selfing nature of beans, which leads to increased amounts of LD. In this study, the cultivars/lines (Andean LD_c/L_ = 4040 Kbp; Meso LD_c/L_ = 428 Kbp) presented slower LD decay compared to the landraces (Andean LD_L_ = 1722 Kbp; Meso LD_L_ = 389 Kbp), consistent with previous studies [31]. The patterns of LD is highly variable among the types of germplasm within species [107], and this variation is determined by several factors, such as the demographic dynamics, recombination rates, and evolutionary mechanisms [108]. The more genetically diverse the germplasm, the more rapid the expected decay, which provides more opportunity for selection, which is extremely important for common bean breeding [109]. The reduced diversity in the cultivated germplasm in Brazil probably is associated with the low maintenance and breeding base population size [22], in addition to the amount of recombination accumulated over the course of selection after breeding programs appeared in the 1930 [22], whereas the landraces have been disseminated by Brazil and domesticated since the sixteenth century [110].

Furthermore, the variation in size of the haplotype blocks across the common bean genome (Table 5) revealed a considerable degree of LD variation, becoming more complex with studies of association and genome selection for beans, as has also been reported for soybean [111]. In this way, the adoption of a general LD value is not recommended, as demonstrated for soybean [107] and wheat [112]. For common bean, it is evident that the level of genetic diversity and LD decay are associated with the germplasm origin and process of domestication, which must be considered to choose the most appropriate strategy for analysis. Considering the total number of haplotypes within the genepools, even with a large number of genotyped markers (5,531), it still seems to be underestimated for the Andean (35.3%) and Mesoamerican (12.2%) gene pools. We clearly observed that these gene pools with distinct process of domestication have to be analyzed independently, using an increased number of SNPs and an effective population size providing a good genetic representativeness to properly define the haplotype databases. The higher the haplotype resolution, the better will be the ability to reconstruct the past gene-flow patterns and to trace key events during the domestication and breeding history [113]. In addition, the haplotypes contribute to the chance of success in identifying regions associated with economic traits, since the associated polymorphism not necessarily have to be the potential causal gene. Similarly, to soybean, a whole-genome analysis, sampling large numbers of markers, will be required, even in selfing crop species with high levels of LD.

### SNP panel

DArTseq analysis over a diverse group of common bean germplasm allowed the identification of a panel composed of 560 SNPs, selected from the whole set of 6286, with nearly 90% genome coverage. For breeding purposes, this panel of SNP, which allows identification of genetic intervals at low to moderate resolution, would be readily incorporated to routine genetic analysis of breeding programs. The benefits of marker-assisted breeding using this panel over a large set of SNPs are due to the increase in the efficiency of genome sampling at a lower cost. This panel certainly will be of great utility for germplasm characterization, linkage mapping, and assisted backcrossing, meeting the research demands with impacts on bean crop systems. For studies that demand improved genome resolution, such as association and genome selection, whole genome sequencing of multiple samples is the method we should use to allow the detection of the majority allelic variants for relevant traits.

## Conclusions

The present study has shown that the DArTseq approach generated a large set of useful SNPs for common bean with a comprehensive genome coverage, representative of coding and non-coding regions that allowed an accurate assessment of structuration and quantification of genetic diversity in the Brazilian core collection composed of landrace and improved germplasm. We also were able to identify genomic regions under selection in domesticated germplasm associated with molecular functions that could be used as target in further studies to determine the nature and relevance of these loci in the process of adaptation. In addition we observed that the extent of LD was variable throughout the genome and in different strata of germplasms, which was helpful for determination of a reduced set of SNPs useful for genetic analysis. Through this study, we are adding value to the common bean Genebank at Embrapa publicly available for worldwide signatory institutions of the International Treaty on Plant Genetic Resources. This information, in combination with phenotypic evaluation, hold much promise for breakthroughs in the elucidation of genetic control of complex traits.

## Additional files

Additional file 1: Identification of the common bean accessions used in the SNP analysis, the gene pool origin, type of germplasm, institution of origin, and the commercial type of grain. (XLSX 18 kb)

Additional file 2: The genotyping data and SNP quality parameters for the whole set of accessions. (XLSX 5705 kb)

Additional file 3: Functional annotation of transcripts affected by SNPs with high (*) or moderate predicted impact. (XLSX 75 kb)

Additional file 4: Enzymes associated with SNP sequences with high and moderate impact predicted. (PDF 22 kb)

Additional file 5: The KEGG pathway maps for SNPs with high or moderate predicted impact. (XLSX 25 kb)

Additional file 6: Gene models representing all SNP with high impact effects predicted by SnpEff. A) High impact effects classified as “splice_acceptor_variant”, defined as two bases before exon start, except for the first exon. B) High impact effects classified as “stop_gained”, a sequence variant whereby at least one base of a codon is changed, resulting in a premature stop codon. The nucleotide described corresponds to the disruptive high impact allele. (PNG 3026 kb)

Additional file 7: Genome-wide loess curves for genetic differentiation (*F*_ST_), Watterson’s θ (θ_W_), and Tajima’s D for all 11 chromosomes in the *P. vulgaris* genome for each group. *F*_ST_ is given as an average across all pairwise comparisons between Andean cultivars/lines and landraces (green), and between Mesoamerican cultivars/lines and landraces (red). The results of Tajima’s D and θ_W_ are given for each group separately, Andean cultivars/lines (dark green) and landraces (green), and Mesoamerican cultivars/lines (red) and landraces (yellow). *F*_ST_, Tajima’s D and θ_W_ related summary statistics were calculated for each 100 kb non-overlapping sliding window. (PDF 240 kb)

Additional file 8: Distribution of SNPs into minor-allele frequency (MAF) classes. (A) Distribution of the number of SNPs into MAF classes for the whole population (grey), Andean (green), and Mesoamerican (red) genotypes. (B) Distribution of the number of SNPs into MAF classes for each group: Andean cultivars/lines (dark green) and landraces (green) and Mesoamerican cultivars/lines (red) and landraces (yellow). (PDF 23 kb)

Additional file 9: Functional annotation of the transcripts affected by outlier SNPs. (XLSX 21 kb)

Additional file 10: Functional annotation showing the most relevant GO terms for the outlier SNPs. The terms were filtered according to the node score. The numbers represent the amount of transcripts related to each term. (PDF 122 kb)

Additional file 11: The KEGG pathway associated with outlier SNPs. (XLSX 15 kb)

Additional file 12: Functional annotation of transcripts affected by SNP outliers in the Mesoamerican gene pool. (XLSX 24 kb)

Additional file 13: Functional annotation of transcripts affected by outlier SNPs in the Andean accessions. (XLSX 15 kb)

Additional file 14: Functional annotation showing the most relevant GO terms for the outlier SNPs within each gene pool. (A) Mesoamerican; (B) Andean. The terms were filtered according to the node score. The numbers represent the amount of transcripts related to each term. (PDF 233 kb)

Additional file 15: Information of the selected 560 SNPs to compose a panel for genetic analysis of common bean. (XLSX 56 kb)

Additional file 16: DAPC analysis based on the 560 SNP panel showing the division between Mesoamerican cultivars/lines (red) and landraces (yellow). M_CL: Mesoamerican cultivars/lines; M_L: Mesoamerican landraces; A_CL (dark green): Andean cultivars/lines; A_L (light green): Andean landraces. (PDF 38 kb)

## Abbreviations

CIAT: International center for tropical agriculture
DAPC: Discriminant analysis of principal components
DArT: Diversity arrays technology
EC: Enzyme code
GBS: Genotyping-by-sequencing
GRM: Genetic relationship matrix
GWAS: Genome-wide association studies
IBGE: Brazilian institute of geography and statistics
IPEF: Institute of forest research and studies
LD: Linkage disequilibrium
LE: Linkage equilibrium
MAF: Minimum allele frequence
MCMC: Markov chain monte carlo
NGS: Next generation sequencing
NJ: Neighboor joining
PIC: Polymorphism information content
QTL: Quantitative trait loci
RAD-seq: Restriction-site-associated DNA sequencing
SIG: Geographic information system
SNPs: Single nucleotide polymorphisms
SSR: Simple sequence repeats
