# Supplementary figures and images for: In-depth genome characterization of a Brazilian common bean core collection using DArTseq high-density SNP genotyping
Source: BMC Genomics. 2017 May 30;18:423. doi: 10.1186/s12864-017-3805-4 (PMC5450071; doi:10.1186/s12864-017-3805-4)

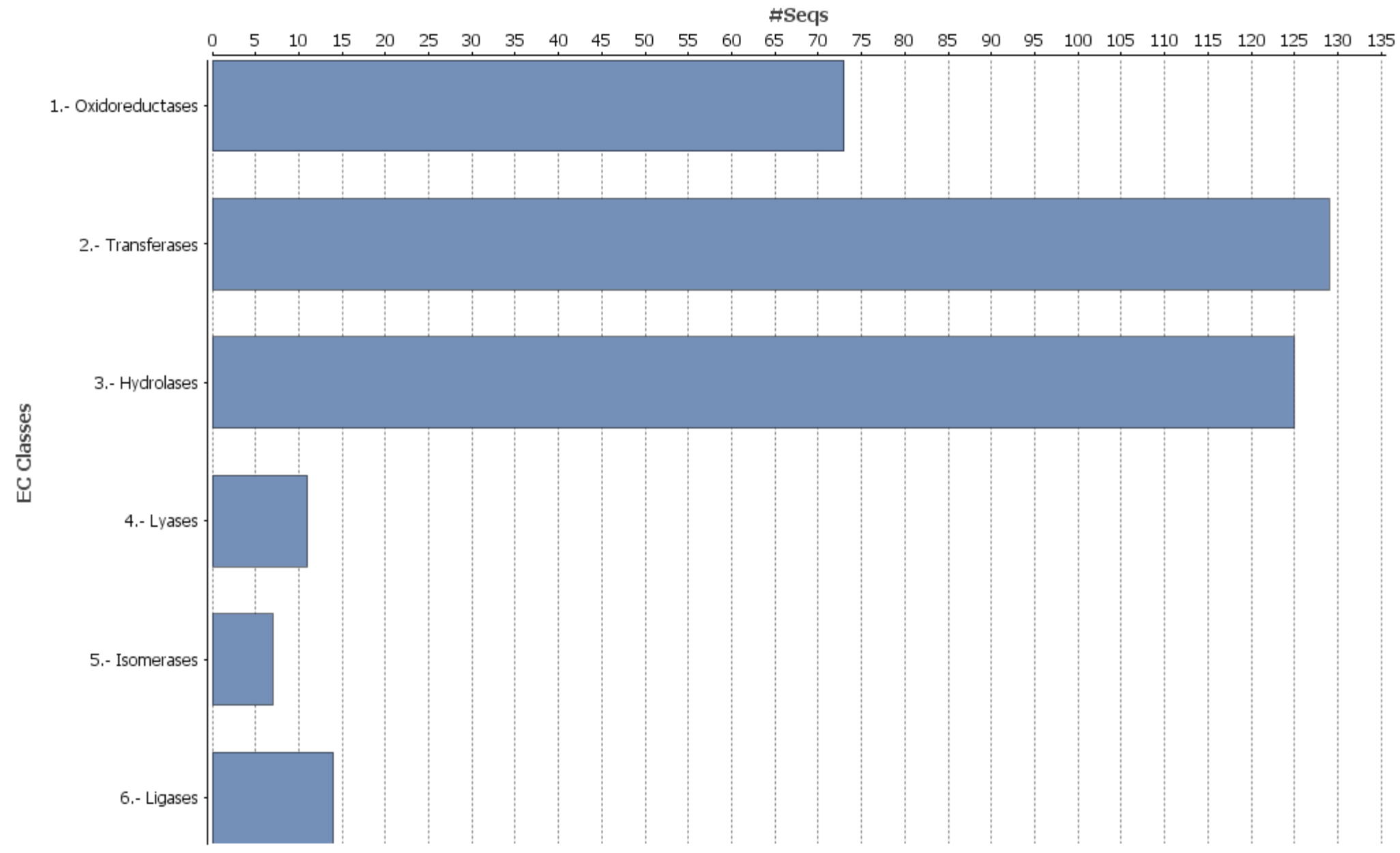

Supplement: Supplementary file 4 — Enzymes associated with SNP sequences with high and moderate impact predicted. (PDF 22 kb) [file 12864_2017_3805_MOESM4_ESM.pdf]

A

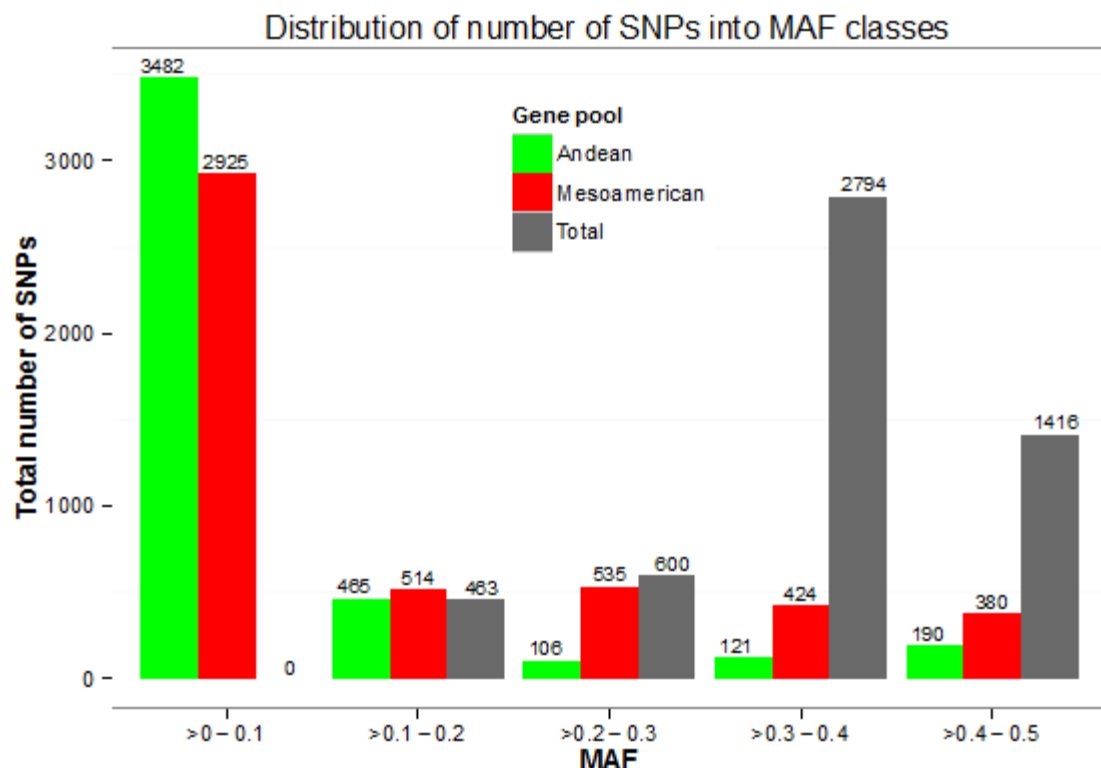

B

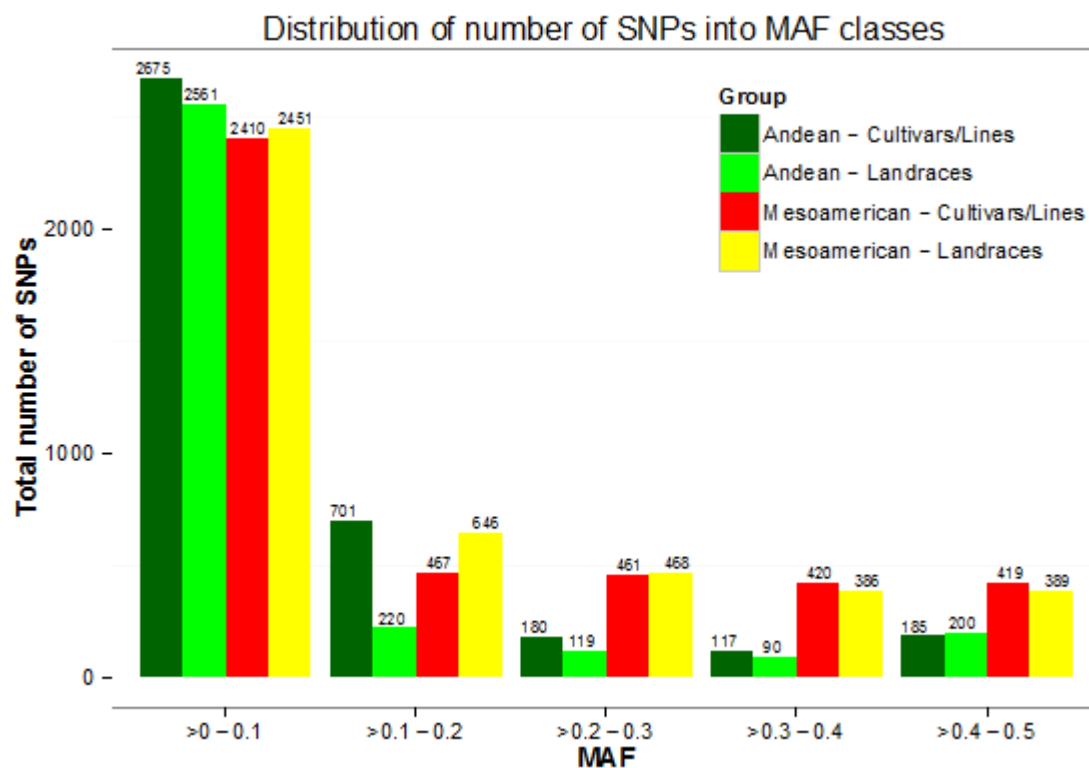

Supplement: Supplementary file 8 — Distribution of SNPs into minor-allele frequency (MAF) classes. (A) Distribution of the number of SNPs into MAF classes for the whole population (grey), Andean (green), and Mesoamerican (red) genotypes. (B) Distribution of the number of SNPs into MAF classes for each group: Andean cultivars/lines (dark green) and landraces (green) and Mesoamerican cultivars/lines (red) and landraces (yellow). (PDF 23 kb) [file 12864_2017_3805_MOESM8_ESM.pdf]

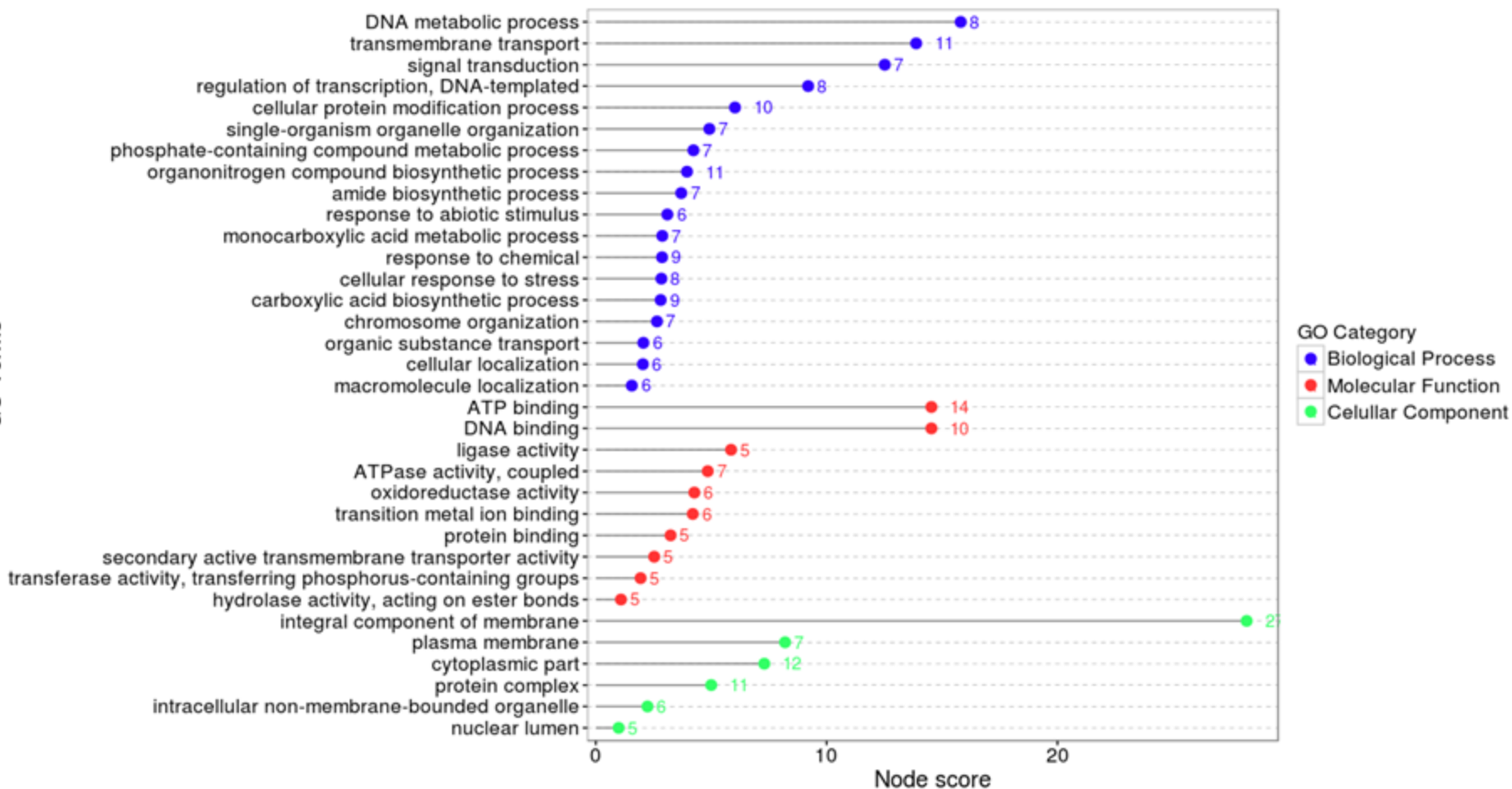

Supplement: Supplementary file 10 — Functional annotation showing the most relevant GO terms for the outlier SNPs. The terms were filtered according to the node score. The numbers represent the amount of transcripts related to each term. (PDF 122 kb) [file 12864_2017_3805_MOESM10_ESM.pdf]

A

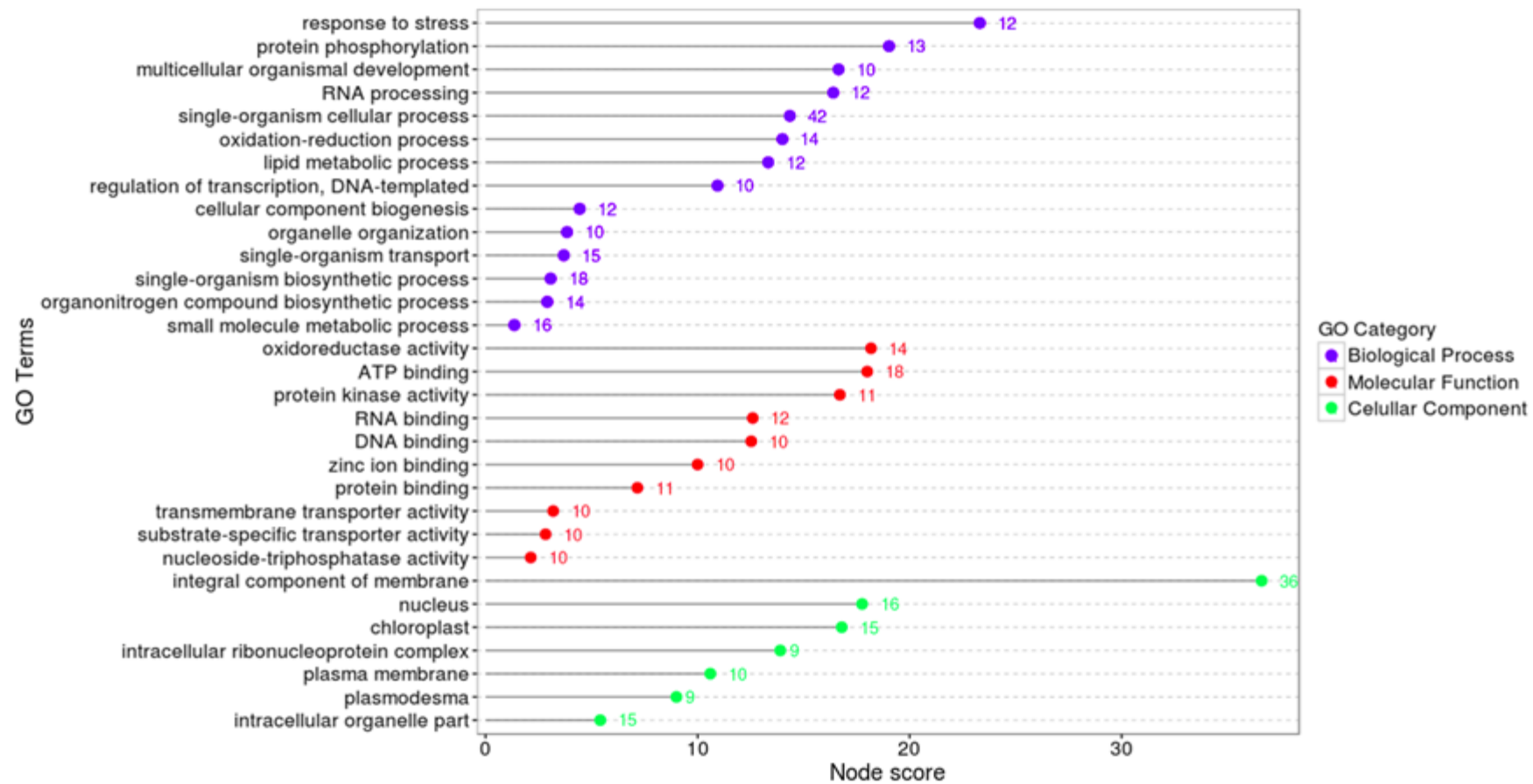

B

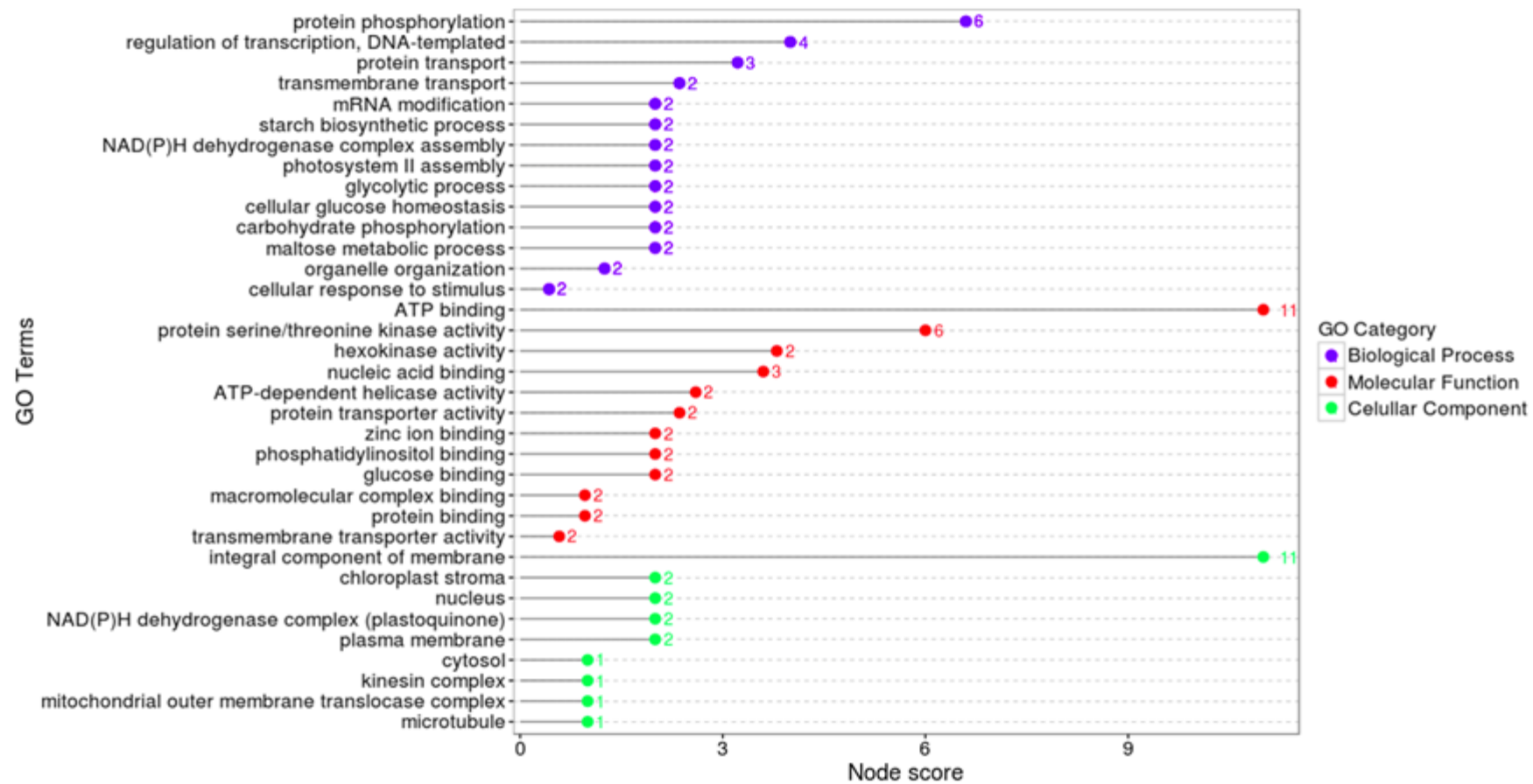

Supplement: Supplementary file 14 — Functional annotation showing the most relevant GO terms for the outlier SNPs within each gene pool. (A) Mesoamerican; (B) Andean. The terms were filtered according to the node score. The numbers represent the amount of transcripts related to each term. (PDF 233 kb) [file 12864_2017_3805_MOESM14_ESM.pdf]

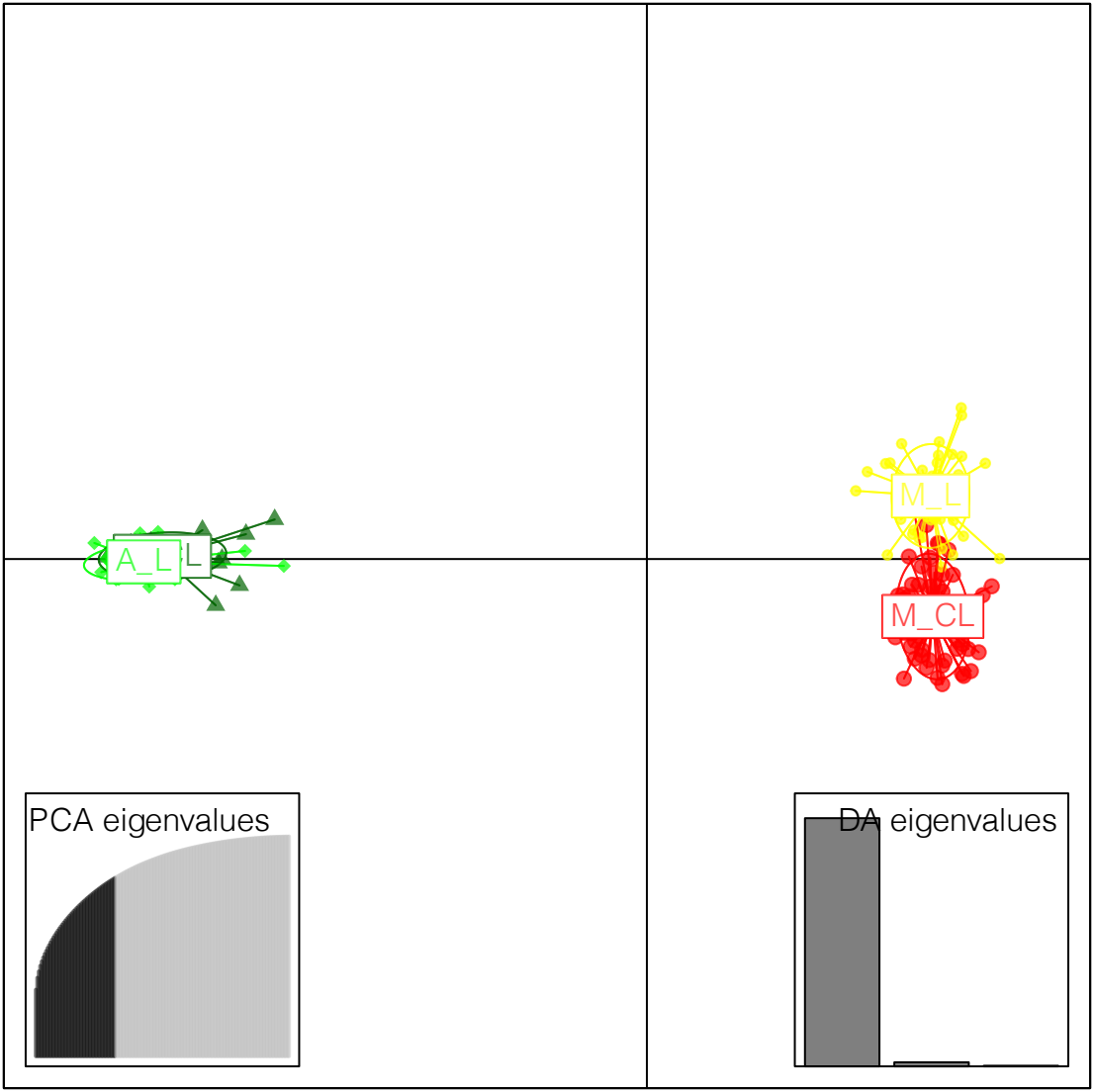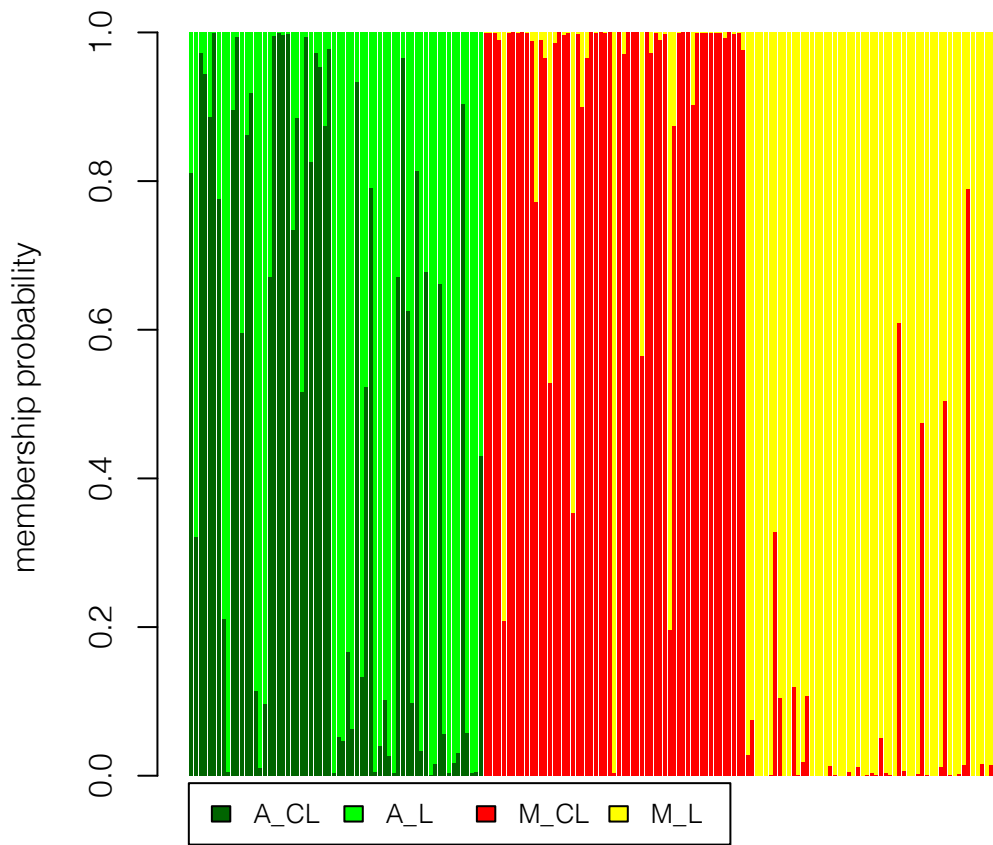

Supplement: Supplementary file 16 — DAPC analysis based on the 560 SNP panel showing the division between Mesoamerican cultivars/lines (red) and landraces (yellow). M_CL: Mesoamerican cultivars/lines; M_L: Mesoamerican landraces; A_CL (dark green): Andean cultivars/lines; A_L (light green): Andean landraces. (PDF 38 kb) [file 12864_2017_3805_MOESM16_ESM.pdf]
