# Supplementary material for: In-depth genome characterization of a Brazilian common bean core collection using DArTseq high-density SNP genotyping
Source: BMC Genomics. 2017 May 30;18:423. doi: 10.1186/s12864-017-3805-4 (PMC5450071; doi:10.1186/s12864-017-3805-4)
Supplement: Supplementary file 7 — Genome-wide loess curves for genetic differentiation (F ST), Watterson’s θ (θW), and Tajima’s D for all 11 chromosomes in the P. vulgaris genome for each group. F ST is given as an average across all pairwise comparisons between Andean cultivars/lines and landraces (green), and between Mesoamerican cultivars/lines and landraces (red). The results of Tajima’s D and θW are given for each group separately, Andean cultivars/lines (dark green) and landraces (green), and Mesoamerican cultivars/lines (red) and landraces (yellow). F ST, Tajima’s D and θW related summary statistics were calculated for each 100 kb non-overlapping sliding window. (PDF 240 kb) [file 12864_2017_3805_MOESM7_ESM.pdf]

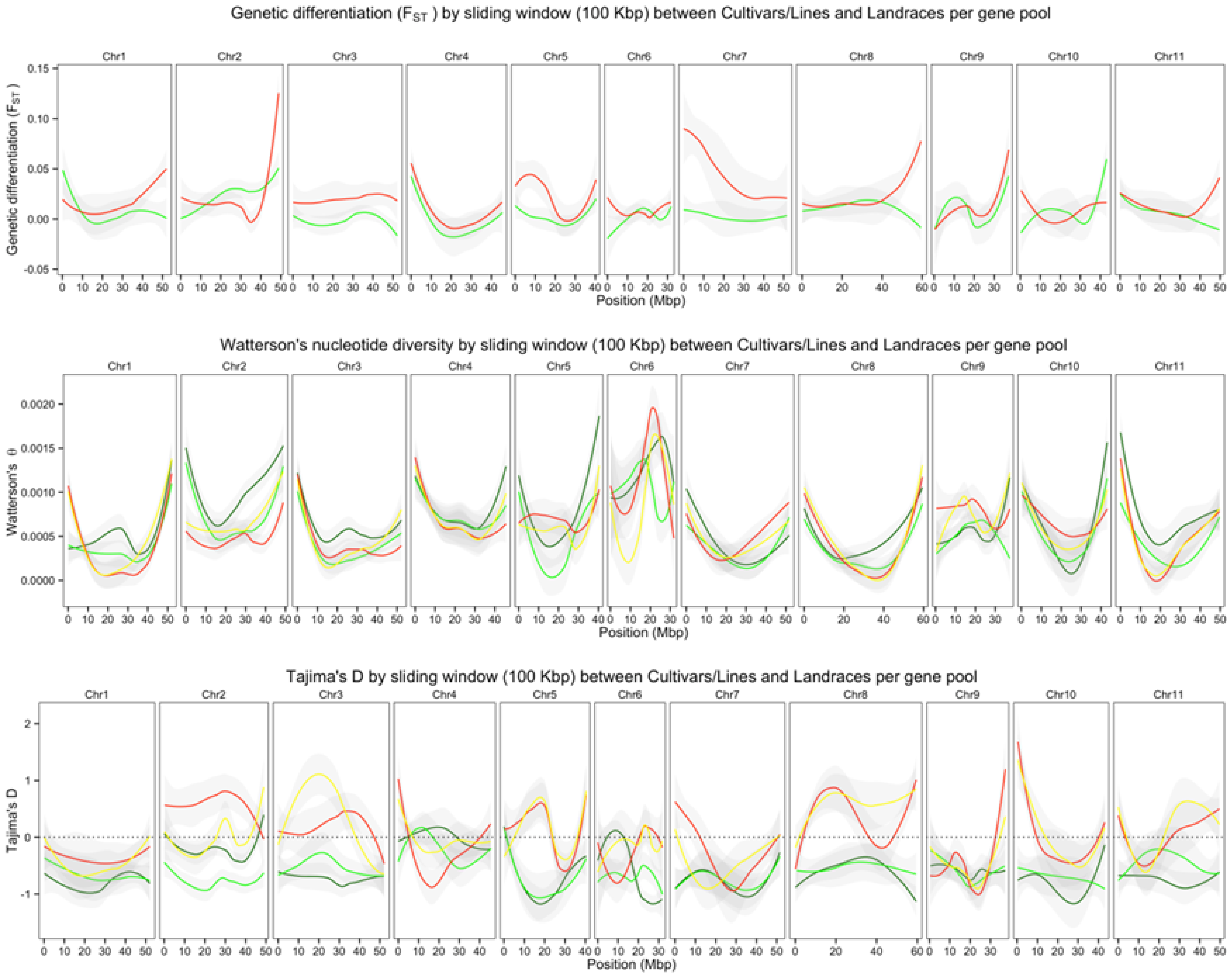
Genetic differentiation (F_{ST}) by sliding window (100 Kbp) between Cultivars/Lines and Landraces per gene pool
Chr1
Chr2
Chr3
Chr4
Chr5
Chr6
Chr7
Chr8
Chr9
Chr10
Chr11
Genetic differentiation (F_{ST})
0.15
0.10
0.05
0.00
-0.05
Position (Mbp)
Watterson's nucleotide diversity by sliding window (100 Kbp) between Cultivars/Lines and Landraces per gene pool
Chr1
Chr2
Chr3
Chr4
Chr5
Chr6
Chr7
Chr8
Chr9
Chr10
Chr11
Watterson's θ
0.0020
0.0015
0.0010
0.0005
0.0000
Position (Mbp)
Tajima's D by sliding window (100 Kbp) between Cultivars/Lines and Landraces per gene pool
Chr1
Chr2
Chr3
Chr4
Chr5
Chr6
Chr7
Chr8
Chr9
Chr10
Chr11
Tajima's D
2
1
0
-1
Position (Mbp)
